# Missense Mutation of POU Domain Class 3 Transcription Factor 3 in *Pou3f3^L423P^* Mice Causes Reduced Nephron Number and Impaired Development of the Thick Ascending Limb of the Loop of Henle

**DOI:** 10.1371/journal.pone.0158977

**Published:** 2016-07-15

**Authors:** Alexandra Rieger, Elisabeth Kemter, Sudhir Kumar, Bastian Popper, Bernhard Aigner, Eckhard Wolf, Rüdiger Wanke, Andreas Blutke

**Affiliations:** 1 Institute of Veterinary Pathology at the Centre for Clinical Veterinary Medicine, Ludwig-Maximilians-Universität München, Munich, Germany; 2 Chair for Molecular Animal Breeding and Biotechnology and Laboratory for Functional Genome Analysis, Gene Center, Ludwig-Maximilians-Universität München, Munich, Germany; 3 Department of Anatomy and Cell Biology, Biomedical Center, Ludwig-Maximilians-Universität München, Munich, Germany; 4 German Center for Diabetes Research (DZD), Helmholtz Zentrum München, Neuherberg, Germany; National Cancer Institute, UNITED STATES

## Abstract

During nephrogenesis, POU domain class 3 transcription factor 3 (POU3F3 *aka* BRN1) is critically involved in development of distinct nephron segments, including the thick ascending limb of the loop of Henle (TAL). Deficiency of POU3F3 in knock-out mice leads to underdevelopment of the TAL, lack of differentiation of TAL cells, and perinatal death due to renal failure. *Pou3f3*^*L423P*^ mutant mice, which were established in the Munich ENU Mouse Mutagenesis Project, carry a recessive point mutation in the homeobox domain of POU3F3. Homozygous *Pou3f3*^*L423P*^ mutants are viable and fertile. The present study used functional, as well as qualitative and quantitative morphological analyses to characterize the renal phenotype of juvenile (12 days) and aged (60 weeks) homo- and heterozygous *Pou3f3*^*L423P*^ mutant mice and age-matched wild-type controls. In both age groups, homozygous mutants *vs*. control mice displayed significantly smaller kidney volumes, decreased nephron numbers and mean glomerular volumes, smaller TAL volumes, as well as lower volume densities of the TAL in the kidney. No histological or ultrastructural lesions of TAL cells or glomerular cells were observed in homozygous mutant mice. Aged homozygous mutants displayed increased serum urea concentrations and reduced specific urine gravity, but no evidence of glomerular dysfunction. These results confirm the role of POU3F3 in development and function of the TAL and provide new evidence for its involvement in regulation of the nephron number in the kidney. Therefore, *Pou3f3*^*L423P*^ mutant mice represent a valuable research model for further analyses of POU3F3 functions, or for nephrological studies examining the role of congenital low nephron numbers.

## Introduction

Members of the POU domain transcription factor family are crucially involved in the regulation of a variety of developmental processes in diverse organs and tissues [1]. Amongst others, the transcription factor POU3F3 (BRN1) plays an important role in the embryonic development of the brain and the kidney, where it participates in regulation of cerebro-cortical neuron migration [2,3] and renal distal tubule formation [4–7]. In the developing brain, POU3F3 acts in concert with another POU domain transcription factor, POU3F2 (BRN2), and loss of function of either one can partially be compensated by the other [2]. However, in the developing kidney only POU3F3 is expressed, largely restricted to parts of the nephron that develop into the thick ascending limb (TAL) and the macula densa (MD) of the loop of Henle and the distal tubule [5].

In the mature kidney, the TAL is functionally important for the urinary concentrating mechanism via the countercurrent multiplier system, the regulation of the extracellular fluid volume and pH, as well as for the homeostasis of calcium and magnesium, and of bicarbonate and ammonium. For this, the TAL is equipped with several, partially energy dependent, specialized ion transporters, such as NKCC2, ROMK, NHE3, CLC-K1/2 and KCC4 [8]. The TAL is also the origin of the abundant urine glycoprotein uromodulin (UMOD), also known as Tamm-Horsfall protein [9,10]. The MD in the juxtaglomerular wall of the distal TAL is crucially involved in regulation of the glomerular blood flow and the glomerular filtration rate (GFR). In response to the intra-tubular sodium chloride concentration, the MD controls the release of renin from juxtaglomerular cells, thereby regulating the renin–angiotensin–aldosterone system (RAAS) dependent tubular reabsorption of sodium and water in the kidneys [11].

In 2003, Nakai and colleagues reported generation of *Pou3f3* knock-out mice [5]. Homozygous knock-outs displayed increased plasma urea and potassium levels and renal hypoplasia with severe malformations of distinct tubular nephron segments. In particular, elongation and differentiation of the TAL and development of MD cells were impaired, and homozygous *Pou3f3* knock-out mice died within 24 hours after birth due to renal failure [5].

Mammalian nephrogenesis is a complex process involving the spatially and timely coordinated interaction of several transcription and growth factors, from initial interaction of the metanephric mesenchyme and the ureteric bud (i.e., nephron induction), to the final formation of the different tubular segments of the mature nephron and the collecting duct (CD) system (i.e., nephron differentiation or nephron patterning) [4,6,7]. In mice, nephron endowment is completed by postnatal day 7–10 [12–14]. The transcription factor POU3F3 is involved in control of this complex nephron patterning, however its role is yet not completely understood, and analysis of POU3F3 function in postnatal renal development in knock-out mice is limited by the neonatal death of the animals.

Recently, we reported generation and phenotypical characterization of a *Pou3f3* mutant mouse line on C3H genetic background in the Munich ethyl-N-nitrosourea (ENU) mouse mutagenesis project [15,16]. These *Pou3f3*^*L423P*^ mutant mice harbor a recessive T→C point mutation, leading to an amino acid exchange from leucine to proline in the conserved homeobox domain of the protein at amino acid position 423 [16]. In contrast to *Pou3f3* knock-out mice, hetero- and homozygous *Pou3f3*^*L423P*^ mutant mice are viable and fertile. They display increased plasma urea levels, reduced body and kidney weights, as well as neurological deficits, impaired hearing, malformation of the semicircular canals of the vestibular organ, and several metabolic changes associated with renal dysfunction [16].

In the present study, the renal phenotype of juvenile and aged homo- and heterozygous *Pou3f3*^*L423P*^ mutant mice was characterized in detail using comprehensive quantitative morphological and functional analyses. State-of-the-art design-based quantitative stereological methods were applied to identify the effects of the *Pou3f3* mutation on development, growth, and cellular composition of different nephron segments, including the TAL and glomeruli. The results of these analyses provide new insights into the role of POU3F3 in pre- and postnatal kidney development and provide the basis for using *Pou3f3*^*L423P*^ mutant mice as an animal model in nephrological research.

## Materials and Methods

### Ethics statement and animal housing

All experiments were approved by the responsible animal welfare authority (Regierung von Oberbayern) and were carried out in accordance with the German Animal Protection Law, conforming to international guidelines on the ethical use of animals.

### Mice

*Pou3f3*^*L423P*^ mutant mice were originally generated in the Munich ENU mouse mutagenesis project and maintained on the genetic background of C3HeB/FeJ inbred mice [16]. All mice investigated in the present study were kept under specified pathogen-free conditions in a closed barrier system on a 12:12 hour light-dark cycle and had free access to a standard rodent diet (V1534; Ssniff, Soest, Germany) and drinking water [16]. Genotyping of mice was performed by allele-specific PCR restriction fragment length polymorphism (RFLP) analysis, as described previously [16]. Mice were examined at 12 days of age and at 60 weeks of age. If not stated otherwise, the following numbers of mice were analyzed at 12 days of age: non-mutant control mice (CON), male: n = 6, female: n = 11; heterozygous *Pou3f3*^*L423P*^*-*mutant mice (HET), male: n = 15, female: n = 15; homozygous *Pou3f3*^*L423P*^-mutant mice (HOM), male: n = 7, female: n = 5; and at 60 weeks of age: CON, male and female: n = 7; HET, male: n = 8, female: n = 7; HOM, male and female: n = 6.

### Blood pressure, serum, and urine analyses

Blood pressure was noninvasively measured by regular tail cuff plethysmography (CODA System, Kent Scientific, Torrington, CT) in 60-week-old mice, as previously described [17]. To this, mice were trained daily to accustom them to the blood pressure measuring procedure over a period of two weeks (at least four times per week). Blood pressure data were averaged from valid readings of ten consecutive measurements per mouse. Spot urine samples from 12-day-old mice and from 60-week-old mice, and serum from tail vein blood specimen from 60-week-old mice were collected and stored at -80°C until assayed. The serum concentrations of urea and sodium were determined using a COBRAS INTEGRA® 400 plus analyzer (Roche Diagnostics, Germany). Serum cystatin C concentrations were measured by an enzyme linked immunosorbent assay (Mouse Cystatin C ELISA-kit RD291009200R, BioVendor GmbH, Germany).

Urine albumin concentrations were determined in spot urine samples of 12-day-old and of 60-week-old mice using the mouse albumin ELISA-kit Bethyl E90-134 (Bethyl, USA). Additionally, the specific gravity of the urine and the urinary creatinine concentrations were determined in spot urine samples of 60-week-old mice. Urine creatinine concentrations were measured using a Hitachi automated analyzer (Merck, Germany). Subsequently, urine albumin-to-creatinine ratios (UACR) were calculated from corresponding urine albumin and creatinine concentrations. The specific gravity of the urine samples was determined by refractometry (MHRS-10-ATC, Müller Optronic, Germany).

For SDS-PAGE urine protein analysis, individual spot urine samples of non-mutant control mice (male: n = 6, female: n = 11), heterozygous *Pou3f3*^*L423P*^-mutant mice (male: n = 15, female: n = 15), and homozygous *Pou3f3*^*L423P*^-mutant mice (male: n = 7, female: n = 5) were diluted to a creatinine content of 1.5 mg/dl. Urine proteins were temperature denatured (Thermoblock TB1, Biometra, Germany) and separated using 12% SDS-PAGE gels (Protean III, Bio-Rad, Munich, Germany) together with a broad molecular weight standard (Bio-Rad) and a mouse albumin standard (Biotrend, Cologne, Germany), as previously described [17]. In all gels, individual urine samples of male and female homozygous and heterozygous *Pou3f3*^*L423P*^-mutant mice and non-mutant controls were loaded. After electrophoresis, the gels were stained with Coomassie blue.

### Body and kidney weight, kidney processing, histology and electron microscopy

After determination of body weight at 12 days and at 60 weeks of age, respectively, mice were killed by cervico-cranial dislocation. After removal of the left kidney, mice were perfused with neutrally buffered 2.5% glutaraldehyde solution through the heart, as previously described [17,18]. After in situ immersion fixation in 2.5% glutaraldehyde solution, the right kidney was carefully separated from adjacent tissues and blotted dry. The kidneys were weighed to the nearest mg and cut perpendicular to the longitudinal axis in ~1 mm thick slices, using a precision tissue slicing device [19]. The slices of the left kidney were fixed in neutrally buffered 4% formaldehyde solution and routinely embedded in paraffin for immunohistochemical analyses.

The slices of the right, perfusion-fixed kidney (6.9 ± 0.5 slices in 12-day-old mice and 11.5 ± 0.9 slices in 60-week-old mice) were routinely processed and embedded in plastic for light microscopy and transmission electron microscopy (TEM) as previously described [20,21]. For TEM, three 1 mm^3^ samples of the renal cortex per animal were selected by systematic random sampling [22], postfixed in 1% osmium tetroxide and embedded in Epon-resin. From each Epon block, ten consecutive 0.5 μm thick semi-thin sections were cut and stained with toluidine blue O and safranin. Ultrathin sections (70–80 nm) were cut, contrasted with uranyl citrate and lead citrate and examined with a TEM (EM10, Zeiss, Eching, Germany). The remaining kidney slices were embedded in plastic, containing hydroxymethylmethacrylate and methylmethacrylate (GMA/MMA, Sigma-Aldrich Laborchemikalien GmbH, Seelze, Germany), as described previously [20]. For qualitative and quantitative morphological analyses, plastic sections with a nominal section thickness of 1.0 μm were cut on a Reichert-Jung 2050 rotary microtome (Leica, Wetzlar, Germany) and stained with hematoxylin and eosin, periodic acid Schiff (PAS), and periodic acid silver methenamine (PASM). For quantitative stereological analyses, ~100 consecutive sections with a nominal section thickness of 1 μm were additionally cut from each GMA/MMA block per case. From each section series, every 20^th^ section was systematically randomly selected (5 ± 1 sections per case) and stained with PAS.

### Immunohistochemistry

Immunohistochemical analyses were performed on sections of paraffin embedded kidney tissue. The following primary antibodies were used: rabbit polyclonal anti human uromodulin antibody (H-135; sc-20631, Santa Cruz Biotechnology, Heidelberg, Germany, dilution 1:1000) [23], monoclonal rat anti mouse uromodulin IgG antibody (MAB5175, R&D Systems, Abingdon, UK, dilution 1:250), polyclonal rabbit anti mouse AQP2 IgG antibody (A7310, Sigma-Aldrich, Munich, Germany, dilution 1:450) and guinea pig anti mouse BRN1 IgG antibody (gp56, by courtesy of Prof. Dr. Wegner, Institute of Biochemistry, Emil Fischer Center, University Erlangen-Nuremberg, Germany, dilution 1:600). As secondary antibodies, biotinylated goat anti rabbit IgG antibody (BA-1000, Vector, Peterborough, UK, dilution 1:200), horseradish peroxidase labeled goat anti rabbit IgG antibody (P0448, DAKO, Hamburg, Germany, dilution 1:150), horseradish peroxidase labeled rabbit anti guinea pig IgG antibody (P0141, DAKO, Hamburg, Germany, dilution 1:150), and alkaline phosphatase labeled goat anti rat IgG antibody (112-055-167, Jackson ImmunoResearch, Newmarket, UK, dilution 1:300) were used. Immunoreactivity was visualized using 3,3-diaminobenzidine tetrahydrochloride dihydrate (DAB) or a combination of nitro blue tetrazolium chloride (NBT) and 5-bromo-4-chloro-3-indolyl phosphate (BCIP). Kidney sections stained with buffer instead of the primary antibody were used as negative control.

### Quantitative-stereological investigations of the kidney

Plastic resin-embedded samples of kidney tissue were used for quantitative stereological analyses, using design-based stereological methods, such as the physical disector and point counting [24,25], essentially carried out as described previously in detail [26]. Quantitative stereological estimates were corrected for embedding-related tissue shrinkage.

#### Total volumes of the kidney, renal zones, glomeruli

The kidney volume was obtained by dividing the weight of the glutaraldehyde-fixed right kidney by the density of perfusion-fixed murine kidney tissue (1.05 g/cm^3^) [27]. The cross-sectional areas of the kidney (Kid) as well as the cortex and the medulla (Med) of the kidney [28,29], were planimetrically determined in micrographs of PASM-PAS stained GMA/MMA kidney sections (9 ± 2 per case), using a Videoplan^TM^ image analysis system (Zeiss-Kontron, Eching, Germany). The volume fractions of the cortex (V_V(Cortex/Kid)_) and the medulla (V_V(Med/Kid)_) were calculated according to the principle of Delesse [24]. The absolute volumes of the individual renal zones (V_(Cortex, Kid)_, V_(Med, Kid)_) were calculated by multiplication of the volume fractions of the respective zones in the kidney with the total kidney volume. The fractional volume of the glomeruli in the cortex (V_V(Glom/Kid)_) was determined by point counting (1029 ± 115 points per case) [30] in systematically randomly selected areas [22] of PASM-PAS stained GMA/MMA sections, as principally described earlier [21]. The absolute volume of the glomeruli in the perfusion-fixed (right) kidney (V_(Glom, Kid)_) was calculated from the volume fraction of the glomeruli in the cortex and the absolute cortex volume. The volume fractions of the TAL in the kidney (V_V(TAL/Kid)_) were determined by point counting (3713 ± 1646 points per case) in systematically randomly selected areas of paraffin embedded kidney sections immunostained for uromodulin (UMOD, section 2.6). The absolute volume of the TAL in the kidney (V_(TAL, Kid)_) was calculated from the volume fraction of the TAL cells in the kidney and the absolute volume of the kidney.

#### Mean glomerular volume and total number of glomeruli

The mean glomerular volume (${\bar{v}}_{\left(\text{Glom}\right)}$) and the total number of nephrons (glomeruli) in the right kidney (N_(Glom, Kid)_) were estimated, using the physical disector method [24,25], in combination with systematic point counting, as described in previous publications of our group [17,21,27]. Per case, 5 ± 1 PAS stained GMA/MMA section pairs with 20 μm distance (disector height) were systematically randomly selected. Using an automated stereology system (VIS-Visiopharm Integrator System^TM^ Version 3.4.1.0 with newCAST^TM^ software, Visiopharm A/S, Denmark), corresponding locations of the renal cortex were then systematically randomly sampled at 40x final magnification in both sections, automatically congruently aligned, and digitally superimposed with unbiased counting frames (344017 μm^2^ area) and 9 x 9 point test grids. The sectional area of cortical kidney tissue present within each sampled counting frame (A_(Cortex)_) was determined by point counting. In the subsequent disector analyses, 67 ± 18 Q^-^ (glomeruli) were counted per case. The numerical volume density of glomeruli in the renal cortex of the right kidney was calculated as: N_V(Glom/Kid)_ = (∑Q^-^_(Glom)_/h x ∑A_(Cortex)_) x f_s_^3^ with f_s_ = linear tissue shrinkage factor for GMA/MMA-embedded murine kidney tissue (0.91) [31]. The number of glomeruli in the right kidney N_(Glom, Kid)_ was then calculated as the product of N_V(Glom/Kid)_ and V_(Cortex, Kid)_. Subsequently, the mean glomerular volume was estimated as ${\bar{v}}_{\left(\text{Glom}\right)}$ = V_V(Glom/Kid)_/ N_V(Glom, Kid)_.

To validate the precise disector heights used for calculation of the numerical volume densities of glomeruli in the kidney and glomerular cells in the glomeruli, the thicknesses of GMA/MMA and Epon sections were controlled, using an orthogonal resectioning technique, as previously described [21,27]. Since the measured thickness of GMA/MMA sections and Epon sections was 1.028 ± 0.031 and 0.501 ± 0.005, respectively, a section thickness of 1.0 μm for GMA/MMA sections and 0.5 μm for Epon sections was consistently used for the calculation of the disector volumes.

#### Number and volumes of glomerular cells

The mean numbers of distinct glomerular cell types per glomerulus (C: all glomerular cells; Pod: podocytes, M-E: mesangial and endothelial cells), and the mean podocyte volume were unbiasedly determined, applying the physical disector method [24,25], principally as described above. For this, the numerical volume density of glomerular cells in the glomerulus was unbiasedly determined in consecutive serial semi-thin sections of Epon-embedded cortical kidney tissue samples: per section series, 2–3 pairs of sections with 1.5 μm distance were systematically randomly sampled [24]. Per case, the corresponding profiles of 10 ± 1 systematically randomly sampled glomeruli were photographed at 400x magnification in both sections of a section pair, using a Leica DFC 320 camera (Leica, Germany) connected to a microscope (Orthoplan, Leitz, Germany). Images including a size ruler were printed and overlaid with a plastic transparency with 576 equally spaced test points. The areas of the glomerular cross-sections (A_(Glom)_) were planimetrically measured, using a Videoplan image analysis system (Zeiss-Kontron, Germany). The volume fraction of podocytes per glomerulus V_V(Pod/Glom)_ was estimated from the fraction of points hitting podocyte section profiles, and points hitting the corresponding glomerular cross-section profile [24]. On the average, 681 ± 206 points were counted per case. All glomerular cell nuclei profiles (C, Pod, M-E) sampled within a glomerular cross-section in the first (reference) section, which were not present in the corresponding glomerular section profile in the second (look-up) section, were counted (Q^−^). The operation was then repeated by interchanging the roles of the look-up section and the reference section, increasing the efficiency of cell nuclei counting by factor two. On the average, 250 ± 47 glomerular cell (C) nuclei (Q^−^) were counted per case (Pod: 72 ± 9; M-E: 181 ± 39). The numerical volume density of glomerular cells in the glomerulus (N_V(C/Glom)_, N_V(Pod/Glom)_, N_V(M-E/Glom)_) was calculated from the number of Q^-^ counted per cell type and the respective disector volume, defined by the cumulative areas of analyzed glomerular section profiles and the distance (disector height = 1.5 μm) between the examined section pairs: N_V(X/Glom)_ = (∑Q^-^_(X)_/h x ∑A_(Glom)_) x f_s_^3^ with X = C, or Pod, or M-E; h = disector height (1.5 μm) and f_s_ = linear tissue shrinkage factor for Epon-embedded murine kidney tissue (0.95) [17]. The mean number of cells per glomerulus (N_(C, Glom)_) and of podocytes per glomerulus (N_(Pod, Glom)_) was calculated by multiplying the numerical volume density of the respective glomerular cells in the glomerulus with the mean glomerular volume. The mean podocyte volume (${\bar{\text{v}}}_{\left(\text{Pod}\right)}$) was calculated dividing V_V(Pod/Glom)_ by N_V(Pod/Glom)_.

#### Volume of the mesangium and glomerular capillaries

The volume densities of capillaries, and of the mesangium within the glomeruli (V_V(Cap/Glom)_ and V_V(Mes/Glom)_) were determined by point counting (fraction of points hitting capillary section profiles, or the mesangial area in PAS stained sections, respectively, and points hitting the glomerular section profile) in 51 ± 6 systematically randomly sampled glomerular profiles in PAS stained GMA/MMA sections at 200x magnification. Per case, 932 ± 256 points were counted. The mean mesangial and capillary volumes per glomerulus (V_(Mes, Glom)_ and V_(Cap, Glom)_) were calculated as the product of the respective volume density in the glomerulus and the mean glomerular volume (${\bar{v}}_{\left(\text{Glom}\right)}$). The total volume of glomerular capillaries in the kidneys was calculated as V_(Glom-cap, Kid)_ = V_V(Cap/Glom)_ x V_(Glom, Kid)_.

#### Length of glomerular capillaries

The mean length of the capillaries per glomerulus (L_(Cap, Glom)_) was determined in the same glomerular profiles sampled in the serial semi-thin Epon sections used for estimation of glomerular cell numbers. The number of glomerular capillary section profiles (ΣQ_(Cap)_ = 1261 ± 122 per case) present within the examined glomerular section profiles was counted. The cumulative glomerular tuft profile area of all examined glomerular section profiles (ΣA_(Glom)_) was measured planimetrically, as described above. The length density of capillaries in the glomeruli was calculated as L_V(Cap/Glom)_ = 2 x Q_A(Cap/Glom)_ x f_s_^2^, with Q_A(Cap/Glom)_ = ΣQ_(Cap)_/ΣA_(Glom)_ and f_s_ = 0.95. L_(Cap, Glom)_ was then obtained as: L_(Cap, Glom)_ = L_V(Cap/Glom)_ x ${\bar{v}}_{\left(\text{Glom}\right)}$ [22,24]. The total length of the glomerular capillaries in the kidney (L_(Glom-cap, Kid)_) was calculated as: L_(Glom-cap, Kid)_ = L_V(Cap, Glom)_ x V_(Glom, Kid)_.

#### Glomerular basement membrane (GBM) thickness and filtration slit frequency (FSF)

The thickness of the GBM was determined by the orthogonal intercept method as described earlier [17,32,33]. Per case, eight glomeruli were sampled from semi-thin sections, as described earlier [34]. Ultrathin sections of these glomeruli were prepared for TEM, and peripheral glomerular capillary loops were photographed (9 ± 2 pictures per case) in a predetermined manner (by half turns of the stage handle). Photographs were developed to a final print magnification of 54324 x, and covered by a transparent 1.5-cm^2^ grid. Where gridlines transected the GBM, the shortest distance between the endothelial cell membrane and the outer lining of the lamina rara externa underneath the cell membrane of the epithelial foot processes was measured, using a logarithmic ruler template with dimensions and midpoints, as described earlier in detail [33]. The true harmonic mean thickness (T_h(GBM)_) of the GBM was estimated as: T_h(GBM)_ = (8/3π) x (10^6^/M) x l_h(GBM)_, with l_h(GBM)_ (apparent harmonic mean GBM thickness) = ∑ Number of observations / ∑ (Midpoints x number of observations), and M = final print magnification. On the average, 1379 ± 264 (range: 938–2204) intercepts per animal were measured. The FSF was determined in the same electron micrographs, by counting the number of epithelial filtration slits divided by the length of the peripheral capillary wall at the epithelial interface, as described earlier [32]. On the average, 1890 ± 667 filtration slits (range: 922–3372) were counted per animal.

### Renal POU3F3 mRNA and protein abundance

#### Quantitative RT-real time PCR (RT-qPCR) analyses

The renal *Pou3f3* mRNA expression abundances in 60-week-old mice were examined by RT-qPCR analyses (n = 7/7 (male/female) non-mutant controls, 8/6 heterozygous mutants, and 5/6 homozygous mutants). RNA-isolation was performed, using Trizol® (Invitrogen, CA, USA). The integrity of RNA was assessed by agarose gel electrophoresis. RNA quantity and purity were determined using a NanoDrop 1000 Spectrophotometer (Thermo Fisher Scientific, Germany). Following DNase digestion (Thermo Scientific dsDNase, Thermo Fisher Scientific, Germany), on the average 2.9 ± 0.4 μg of total RNA per case were reverse transcribed using the RNA to cDNA EcoDryTM Premix (Clontech, CA, USA). RT-qPCR analyses were performed on an Applied Biosystems StepOne Real-Time PCR System (Applied Biosystems, Germany) using Power SYBR^®^ Green PCR Master Mix (Applied Biosystems, UK) and primers for amplification of mouse *Pou3f3* [35] (accession N°: NM_008900.2, forward primer: 5’-GGT ACC CAC CTG CGA GTA GA-3’; reverse primer: 5’-CAG CCT ACA GCT GGA AAA GG-3’; amplicon length: 127 bp), *uromodulin (Umod)* [36] (accession N°: NM_001278605.1, forward primer: 5’-gga aag cag aaa acc tgg tg-3’; reverse primer: 5’-gag aca ggg ctt cat aca-3’; amplicon length: 205 bp), and *glyceraldehyde-3-phosphate dehydrogenase (Gapdh)* [37] (accession N°: NM_001289726.1, forward primer: 5’-TGT GTC CGT CGT GGA TCT GA-3’; reverse primer: 5’-CCT GCT TCA CCA CCT TCT TGA T-3’; amplicon length: 77 bp). Primer sequences were queried by NCBI Blast software and the comparability of amplification efficiencies of the single primers was confirmed by performance of RT-qPCR-based standard curve analyses. All RT-qPCR measurements were performed in duplicates and included no template controls and RT-minus controls (DNase-digested RNA). The expression abundances of *Pou3f3*, and *Umod* were calculated in relation to the expression of *Gapdh* as internal reference, as well as the abundance of *Pou3f3* in relation to *Umod* using the 2^-ΔCT^ method [38]. The relative mRNA abundances were then normalized to the respective mean relative mRNA abundance of non-mutant control mice.

#### Western-blot analyses

Renal protein abundances of POU3F3 and GAPDH in 60-week-old mice were examined by Western-blot analyses (n = 6/6 (male/female) non-mutant controls, 6/6 heterozygous mutants, and 6/6 homozygous mutants). For protein extraction, kidney tissue samples were homogenized in protein extraction buffer (10 mm Na_2_HPO_4_ at pH 7.0; 0.2% (wt/vol) sodium dodecyl sulfate solution; 10% (vol/vol) glycerine; adjusted to pH 7.0), heated to 95°C for 5 minutes and cooled on ice for 5 minutes. After centrifugation (18,000 g, 5 min, 4°C), protein contents were quantified using the bicinchoninic acid method [39]. For Western-blot analyses, 30 micrograms of denatured protein were loaded per lane on 12% sodium dodecyl sulfate-polyacrylamide gels and separated by electrophoresis (SDS–PAGE) together with a prestained protein ladder (PageRuler, Thermo Scientific, Germany). After electrophoresis, separated proteins were blotted to polyvinylidene difluoride (PVDF) membranes (Thermo Fisher Scientific, Germany). For detection of POU3F3 and GAPDH, the following antibodies (diluted in 5% dry milk) were used after blocking of the membranes: Guinea pig-anti-Brn1 antibody, diluted 1:2500 [40]; and Rabbit-anti-GAPDH (D16H11) mAb (#51741, Cell Signaling, New England Biolabs GmbH, Germany), diluted 1:2000. Incubation with primary antibodies was carried out overnight at 4°C. After washing of the membranes, incubation with appropriate horseradish-peroxidase coupled secondary antibodies (HRP-goat anti-rabbit IgG (#7074), Cell Signaling, New England Biolabs GmbH, Germany), diluted 1:2000; Donkey anti-guinea pig IgG HRP conjugated (43R-ID039hrp, Fitzgerald Industries International Inc., Bioleague GmbH & Co.KG, Germany), diluted 1:5000) and final washing steps, bound antibodies were visualized using Amersham ECL Western blotting detection reagent and Amersham ECL Hyperfilms (GE Healthcare Life Sciences, Germany). After detection of POU3F3, the membranes were stripped in stripping buffer (37.5 ml of 250 mM TRIS buffer at pH 6.7; 30 ml of 10%; 82.5 ml H2O; 1050 μl ß-mercaptoethanol) for 40 minutes at 70°C and then used for detection of GAPDH.

### Statistical analyses

All data are presented as means ± SD. To determine genotype effects data were analyzed by one-way ANOVA with Gabriel’s post hoc tests. Gender specific differences were analyzed by comparison of male *vs*. female mice of identical genotypes by using an unpaired Student's t-test (IBM SPSS Statistics, Version 18). P-values <0.05 were considered significant. For all examined parameters, the exact p-values of all comparisons are provided in the Supporting Information (S1 and S2 Tables).

## Results

### Body weights, absolute and relative kidney weights, and kidney volumes

**Fig 1:**
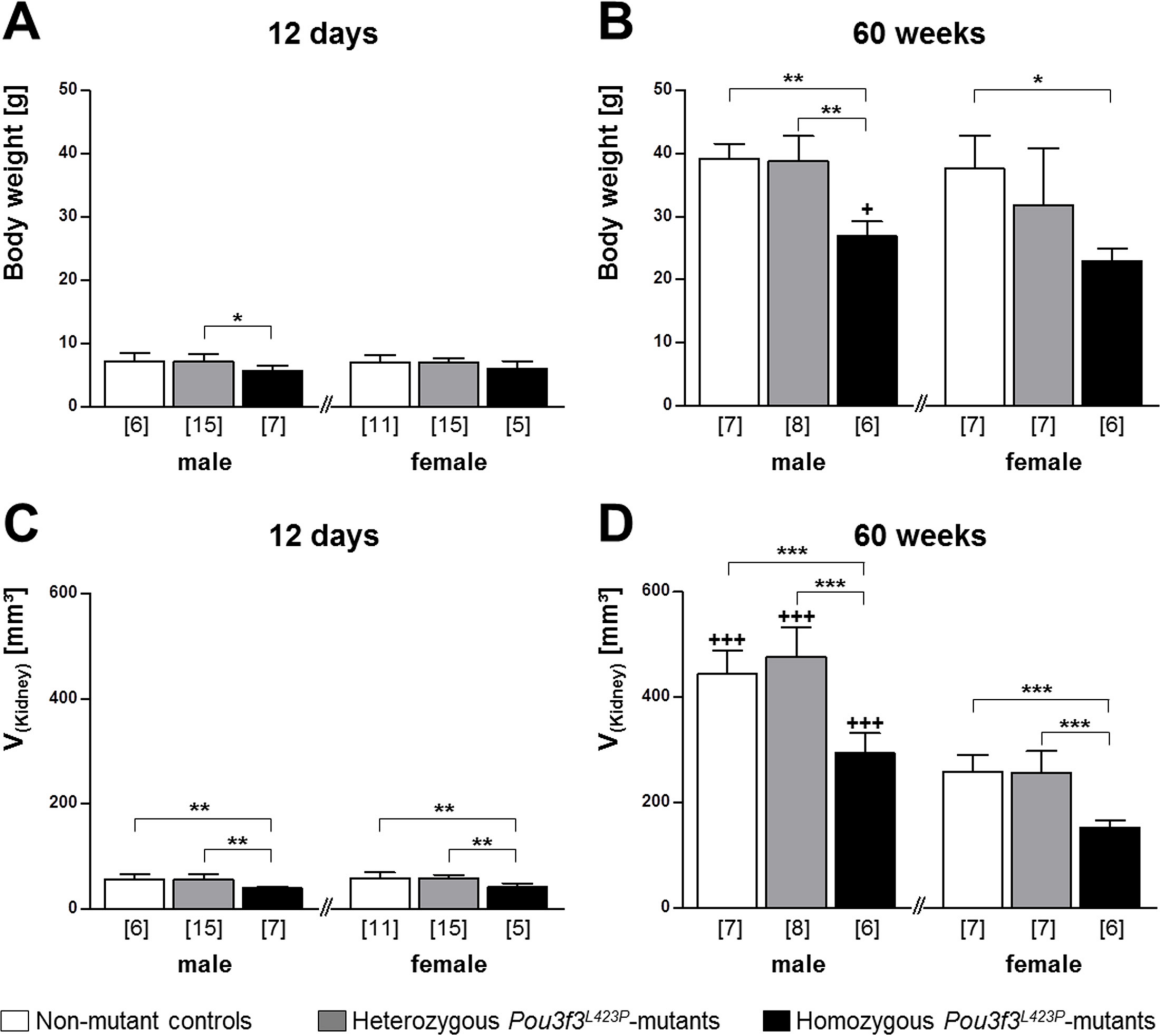
**Body weight (A, B) and volume of the perfusion-fixed right kidney (C, D) of 12-day-old (A, C) and of 60-week-old (B, D) *Pou3f3***^***L423P***^ **mutant mice and non-mutant control mice.** The numbers of animals examined are given in parentheses. Data are means ± SD. Significant differences between homozygous and heterozygous mutants and non-mutant control mice are indicated by asterisks. *: p < 0.05, **: p < 0.01, ***: p < 0.001. Significant differences between male and female mice of identical genotypes are indicated by crosses. +: p < 0.05, ++: p < 0.01, +++: p < 0.001.

In juvenile mice of 12 days of age, the body weights of male and female homozygous *Pou3f3*^*L423P*^ mutant mice tended to be reduced as compared to heterozygous mutants and control mice (Fig 1), reaching statistical significance in male homozygous vs. heterozygous mutants (p<0.05). Homozygous *Pou3f3*^*L423P*^-mutant mice consistently displayed significantly lower absolute (p<0.01) and relative kidney weights (p<0.05) and kidney volumes (p<0.01) than heterozygous mutants or non-mutant control mice. However these parameters were not significantly different between heterozygous mutants and control mice (Table 1, Fig 1). At 60 weeks of age, the body weights of male homozygous *Pou3f3*^*L423P*^ mutant mice were significantly smaller, as compared to heterozygous mutants (male p<0.01) or non-mutant control mice (male p<0.01, female p<0.01). Furthermore the reduction of the absolute kidney weights, as well as the kidney volumes of homozygous *Pou3f3*^*L423P*^ mutant mice of both sexes was highly significant (p<0.001). In contrast, the body weights, absolute and relative kidney weights, and kidney volumes were not significantly different between heterozygous mutants and non-mutant control mice (Table 2, Fig 1).

**Table 1:** Absolute and relative kidney weights and urine albumin concentrations in homozygous (HOM) and heterozygous (HET) *Pou3f3*^*L423P*^ mutant mice and control (CON) mice at 12 days of age.

Parameter	Sex	CON *(6/11)*		HET *(15/15)*		HOM *(7/5)*		Statistical significance*		
								*CON vs*. *HET*	*CON vs*. *HOM*	*HET vs*. *HOM*
Kidney weight [mg]	M	63	±11	60	±11	42	±3	*n*.*s*.	*p<0*.*01*	*p<0*.*01*
	F	62	±11	62	±8	46	±8	*n*.*s*.	*p<0*.*01*	*p<0*.*01*
Relative kidney weight [% of body weight]	M	1.8	±0.2	1.7	±0.1	1.5	±0.1	*n*.*s*.	*p<0*.*05*	*p<0*.*05*
	F	1.8	±0.1	1.8	±0.2	1.5	±0.1	*n*.*s*.	*p<0*.*05*	*p<0*.*05*
Urine albumin concentration [μg/ml]	M	25	±9	18	±6	18	±8	*n*.*s*.	*n*.*s*.	*n*.*s*.
	f	19	±3	21	±7	15	±4	*n*.*s*.	*n*.*s*.	*n*.*s*.

Numbers of animals examined are given in brackets (male/female). Data are means ± SD.

* 1-way ANOVA with Gabriel’s post hoc test. *n*.*s*. not significant.

**Table 2:** Absolute and relative kidney weights, serum sodium levels, and urine albumin and creatinine concentrations in homozygous (HOM) and heterozygous (HET) *Pou3f3*^*L423P*^ mutant mice and control (CON) mice at 60 weeks of age.

Parameter	Sex	CON *(7/7)*		HET *(8/7)*		HOM *(6/6)*		Statistical significance*		
								*CON vs*. *HET*	*CON vs*. *HOM*	*HET vs*. *HOM*
Kidney weight [mg]	m	365 ^c^	±23	399 ^b^	±55	229 ^b^	±31	*n*.*s*.	*p<0*.*001*	*p<0*.*001*
	f	198 ^c^	±23	219 ^b^	±33	112 ^b^	±11	*n*.*s*.	*p<0*.*001*	*p<0*.*001*
Relative kidney weight [% of body weight]	m	0.9 ^c^	±0.1	1.0 ^a^	±0.1	0.9 ^b^	±0.1	*n*.*s*.	*n*.*s*.	*p<0*.*05*
	f	0.5 ^c^	±0.1	0.7 ^a^	±0.2	0.5 ^b^	±0.0	*n*.*s*.	*n*.*s*.	*p<0*.*05*
Serum sodium concentration [mmol/l]	m	146	±5	146	±4	147	±4	*n*.*s*.	*n*.*s*.	*n*.*s*.
	f	141	±6	143	±1	144	±10	*n*.*s*.	*n*.*s*.	*n*.*s*.
Urine albumin concentration [μg/ml]	m	50	±20	57	±22	25	±24	*n*.*s*.	*n*.*s*.	*p<0*.*05*
	f	125	±81	38	±22	11	±8	*n*.*s*.	*p<0*.*01*	*n*.*s*.
Urine creatinine concentration [mg/ml]	m	0.21	±0.05	0.16	±0.05	0.14	±0.11	*n*.*s*.	*n*.*s*.	*n*.*s*.
	f	0.19	±0.04	0.16	±0.04	0.10	±0.03	*n*.*s*.	*p<0*.*05*	*n*.*s*.
UACR [μg/mg]	m	253.3	±67.0	337.8	±121.9	183.9	±48.6	*n*.*s*.	*n*.*s*.	*n*.*s*.
	f	642.4	±433.3	258.5	±140.1	114.9	±76.7	*n*.*s*.	*p<0*.*05*	*n*.*s*.

Numbers of animals examined are given in brackets (male/female). UACR: Urine albumin-to-creatinine ratio. Data are means ± SD.

*1-way ANOVA with Gabriel’s post hoc test.

^a,b,c^: Statistically significant differences (a: p≤0.05, b: p≤0.01, c: p≤0.001) between male and female mice of the identical genotype; *n*.*s*. not significant.

### Blood pressure, serum, and urine analyses

**Fig 2:**
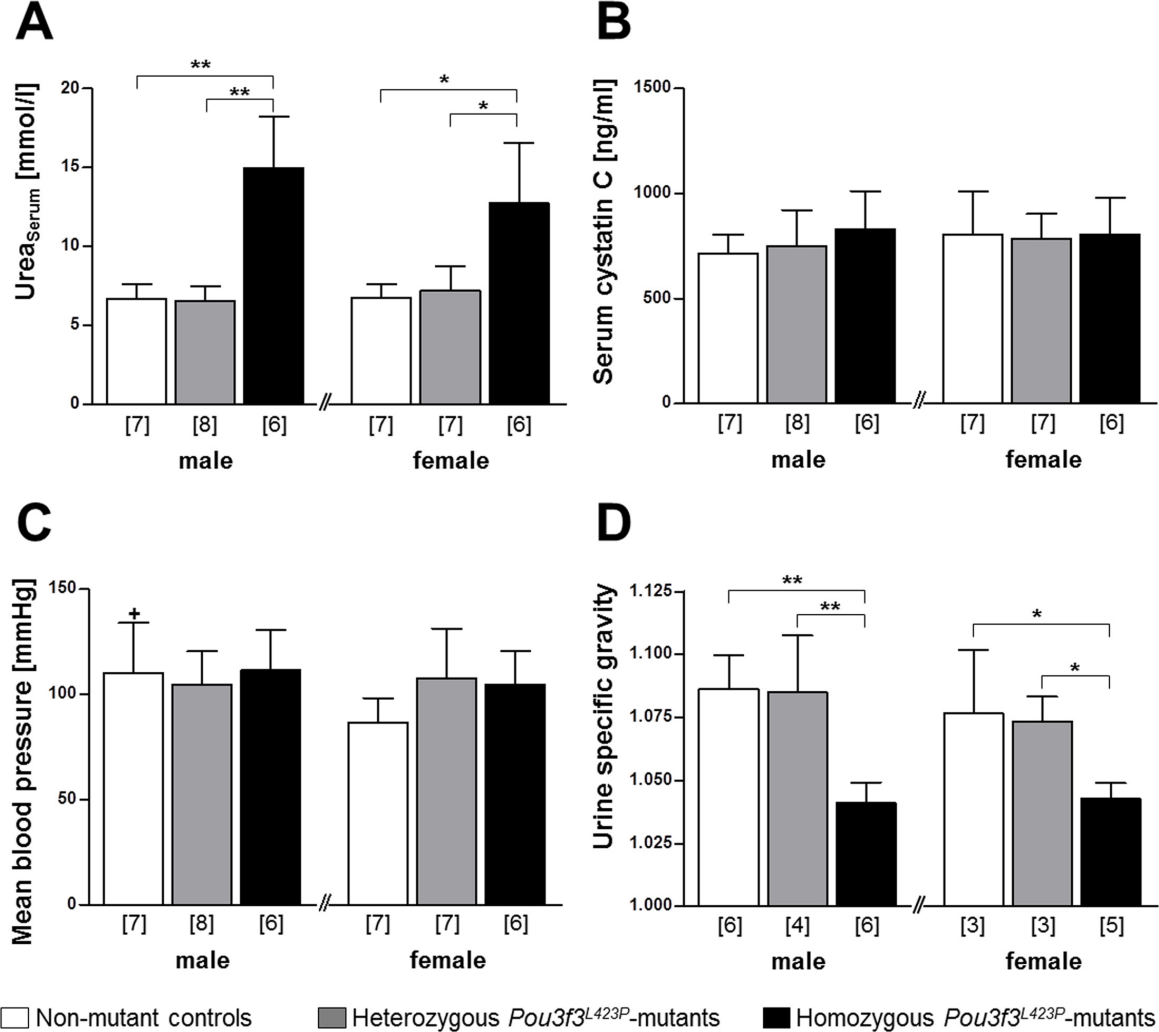
**Serum urea concentration (A), serum cystatin C concentration (B), mean blood pressure (C), and urine specific gravity of 60-week-old *Pou3f3***^***L423P***^ **mutant mice and non-mutant control mice (D).** The numbers of animals examined are given in parentheses. Data are means ± SD. Significant differences between homozygous and heterozygous mutants and non-mutant control mice are indicated by asterisks. *: p < 0.05, **: p < 0.01, ***: p < 0.001. Significant differences between male and female mice of identical genotypes are indicated by crosses. +: p < 0.05, ++: p < 0.01, +++: p < 0.001.

The mean blood pressures of 60-week-old homo- and heterozygous *Pou3f3*^*L423P*^ mutants and of non-mutant control mice of the same sex did not display statistically significant differences. Comparing male and female mice of identical genotypes, however, male non-mutant control mice displayed significantly higher (p<0.05) mean blood pressures than female mice (Fig 2).

For characterization of the renal function in 60-week-old mice, the serum concentrations of urea, sodium, and cystatin C, as well as the urine concentrations of albumin and creatinine, and the specific gravities of spot urine samples were determined. Homozygous mutants displayed significantly, on average two-fold higher serum urea concentrations than non-mutant control mice and heterozygous mutants (male mice: p<0.01, female mice: p<0.05), whereas the serum urea levels between heterozygous mutants and control mice were not significantly different (Fig 2A).

The serum concentrations of sodium (Table 2) and of cystatin C (Fig 2B), an endogenous marker of glomerular filtration [41], were also not significantly different between the different groups of mice.

**Fig 3:**
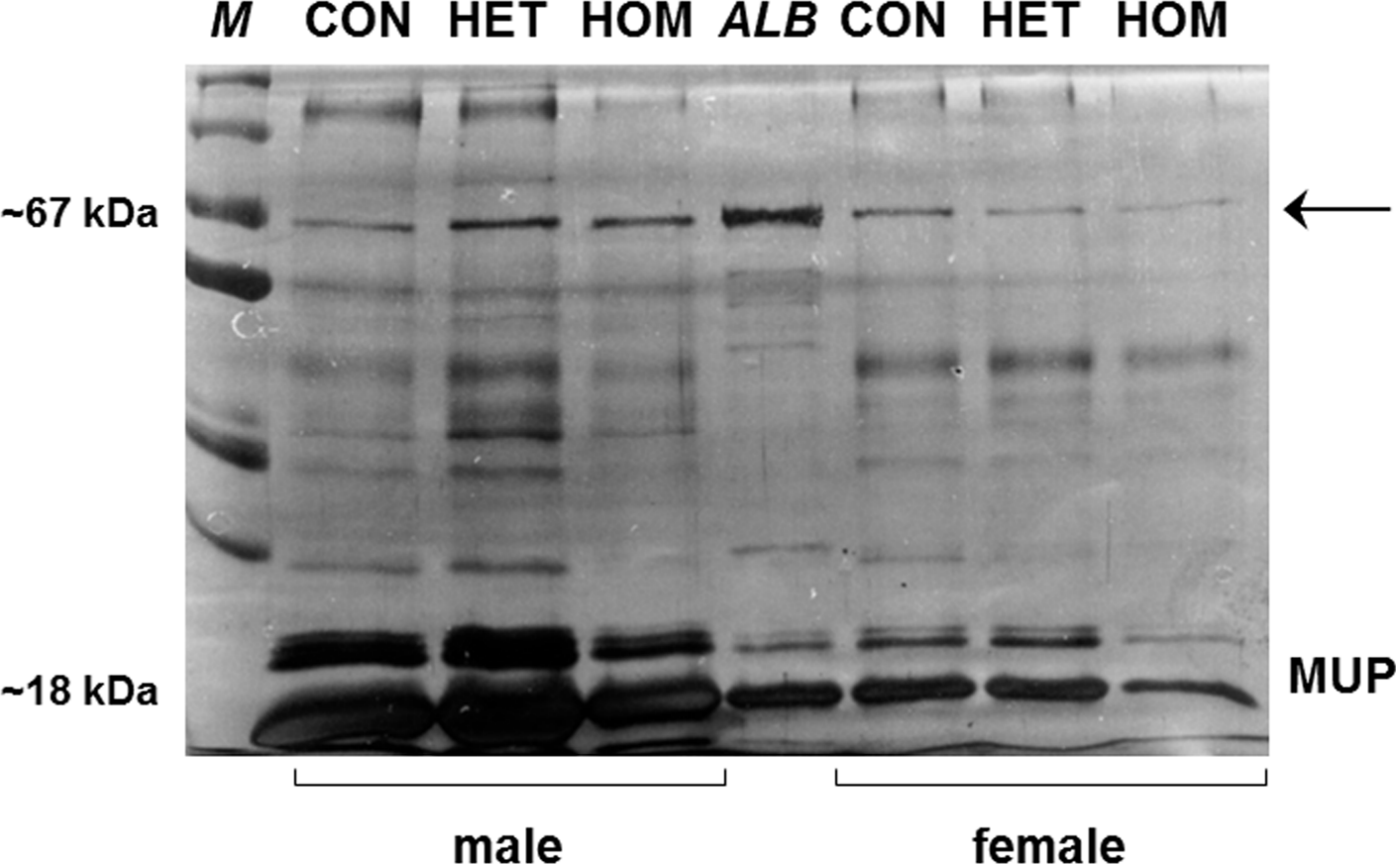
SDS-PAGE urine protein analysis of 60-week-old homozygous (HOM) and heterozygous (HET) *Pou3f3*^*L423P*^ mutant mice and non-mutant control (CON) mice. A representative gel of individual spot urine samples of three male and female HOM, HET and CON mice diluted to identical creatinine concentrations is shown. Molecular weight marker (M), murine serum albumin (ALB). Mice of all examined genotypes display discrete albumin bands (arrow) at approximately 67 kDa. The intensity of the major urinary protein (MUP) bands at approximately 18–25 kDa in male mice is stronger than in female mice.

Urine analyses results revealed no evidence of albuminuria in *Pou3f3*^*L423P*^ mutant mice. At 12 days of age, the urine albumin concentrations were not significantly different between homo- and heterozygous Pou3f3^L423P^ mutants and control mice (Table 1). At 60 weeks of age, the urine albumin concentrations were even significantly lower in female homozygous mutants *vs*. non-mutant controls (p<0.01), as well as in male homo- *vs*. heterozygous mutants (p<0.05) (Table 2). Moreover, 60-week-old homozygous *Pou3f3*^*L423P*^ mutant mice also consistently displayed lower urine creatinine concentrations, urinary albumin-to-creatinine ratios, and urine specific gravities than heterozygous *Pou3f3*^*L423P*^ mutants and non-mutant control mice. Here, the specific urine gravity (Fig 2D) in homozygous mutants was significantly lower than in heterozygous mutants and control mice (male mice: p<0.01, female mice: p<0.05). as well, the urine creatinine concentrations and the urine albumin-to-creatinine ratios (UACR) (Table 2) of female homozygous mutants were also statistically significantly lower as compared to control mice (p<0.05). In contrast, the specific urine gravities (Fig 2D), the urine creatinine concentrations and the UACRs (Table 2) were not significantly different between heterozygous mutants and non-mutant controls mice (Fig 2). Corresponding to these quantitative findings, SDS-PAGE based urine protein analyses did also not demonstrate signs of albuminuria in homo- or heterozygous *Pou3f3*^*L423P*^ mutants (Fig 3).

### Qualitative histological, immunohistochemical and ultrastructural kidney findings

**Fig 4:**
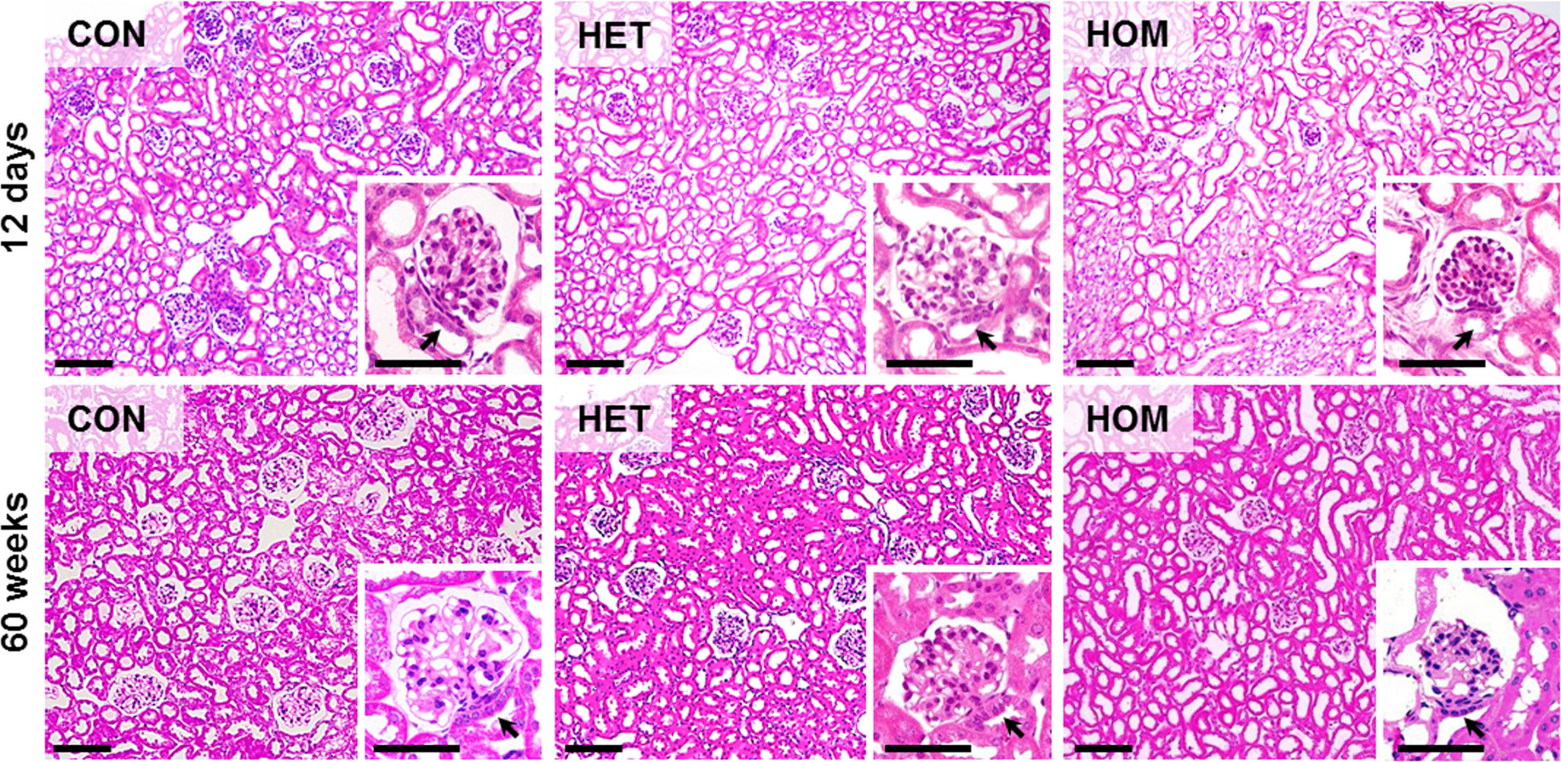
Kidney histology of 12-day-old and of 60-week-old homozygous (HOM) and heterozygous (HET) *Pou3f3*^*L423P*^ mutant mice and non-mutant control mice (CON). GMA/MMA sections, HE staining. Bars = 100 μm, in insets = 50 μm.

Histological evaluation revealed an obvious decrease in the number of glomerular profiles in kidney sections of 60-week-old homozygous *Pou3f3*^*L423P*^ mutant mice, as compared to heterozygous mutants and non-mutant control mice. Moreover, the glomerular section profiles of homozygous *Pou3f3*^*L423P*^ mutants appeared strikingly smaller than in heterozygous *Pou3f3*^*L423P*^ mutants or control mice (Fig 4). In contrast, histological examination of kidney sections revealed no conspicuous differences between heterozygous *Pou3f3*^*L423P*^ mutants and control mice (Fig 4).

**Fig 5:**
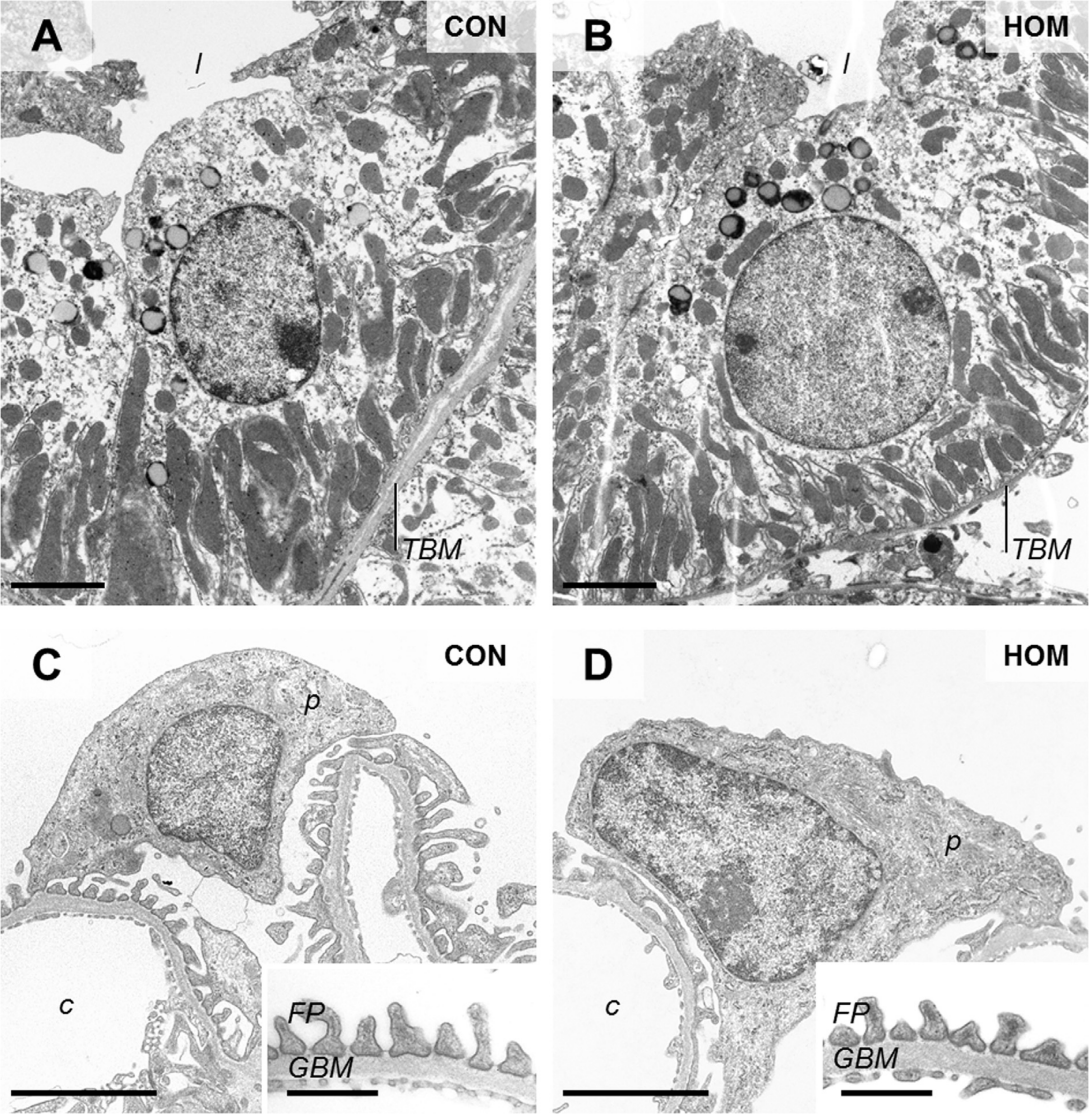
**Ultrastructural morphology of TAL cells (A, B), podocytes and peripheral glomerular capillary walls (C, D) in 60-week-old, male, homozygous *Pou3f3***^***L423P***^ **mutant mice (HOM) and non-mutant control mice (CON).** Tubular lumen (*l*), tubular basement membrane (*TBM*), podocyte (*p*), glomerular capillary (*c*), podocyte foot processes (*FP*), glomerular basement membrane (*GBM*), Transmission electron microscopy. Bars = 2.5 μm A-D, and = 0.5 μm in insets to C, D.

Apart from the apparently diminished size of glomeruli in 60-week-old homozygous *Pou3f3*^*L423P*^ mutants, which was more prominent than in 12-day-old mutant mice, no qualitative histopathological or ultrastructural glomerular alterations were present in either homo- or heterozygous mutants at 12-days and at 60-weeks of age (Figs 4 and 5). Histological and ultrastructural analysis also showed no conspicuous morphological alterations of the epithelial cells of the thick ascending limb (TAL) of the loop of Henle of 12-day-old, or of 60-week-old homo- and heterozygous *Pou3f3*^*L423P*^ mutant mice (Figs 4 and 5).

**Fig 6:**
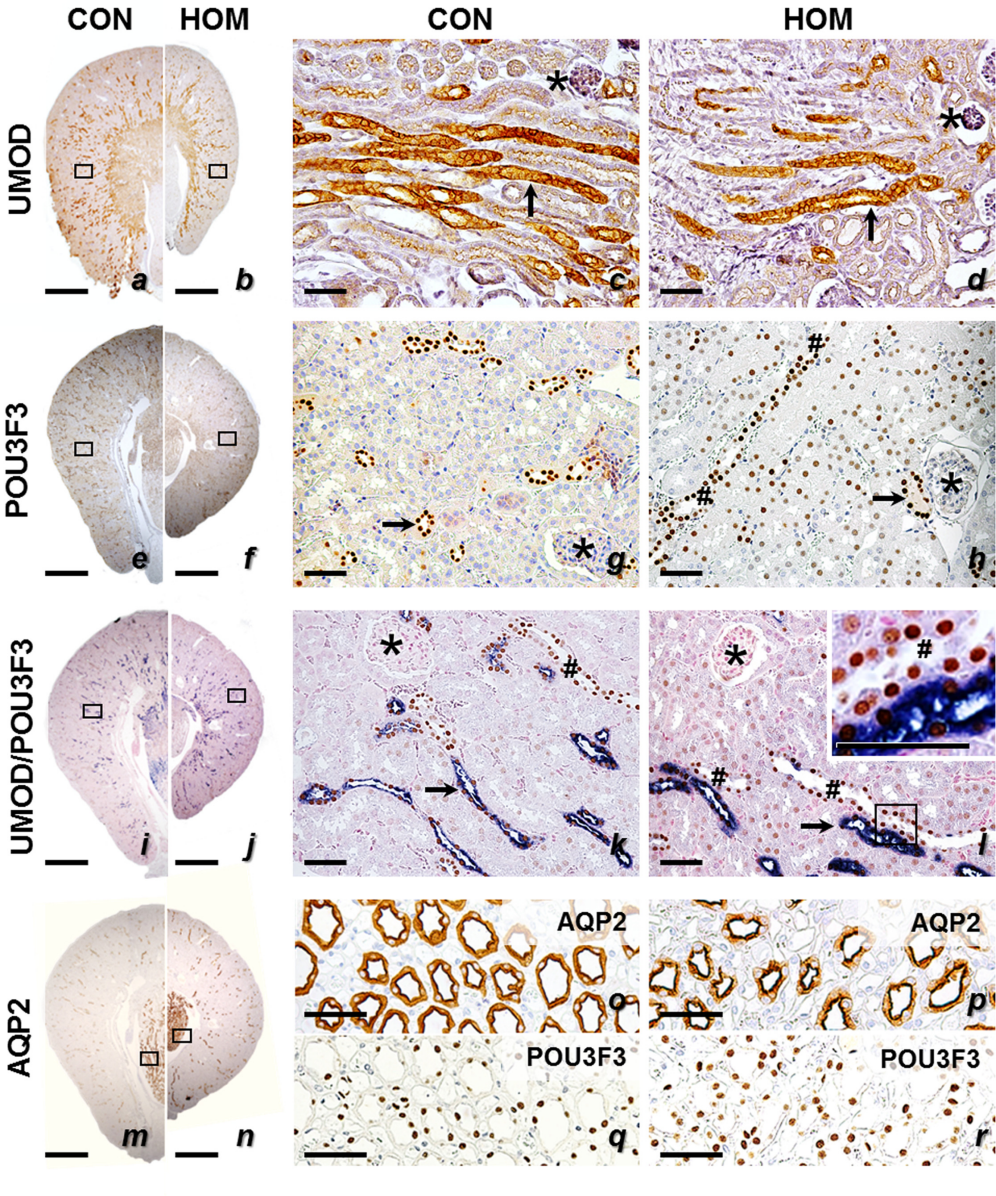
**Immunohistochemical detection of uromodulin (UMOD, *a-d*), POU3F3 (*e-l*), and Aquaporin 2 (AQP2, *m-r*) in kidney sections of 60-week-old homozygous *Pou3f3***^***L423P***^ **mutant mice (HOM) and non-mutant control mice (CON).** Positive immunoreactivity (UMOD, POU3F3, AQP2 in a-h and m-r) is indicated by brown color (chromogen: DAB). In the double-immunohistochemical detection (i-l), UMOD immunoreactivity is indicated by blue color (chromogen: NBT/BCIP) and POU3F3 by brown color (DAB). Arrows mark the TAL and the MD, CDs are indicated by hashs, and asterisks mark glomeruli. Black rectangles indicate the positions of the detail enlargements. Positive UMOD immunoreactivity with a diffuse cytoplasmic and distinct apical membrane staining pattern is present in epithelial cells of the TAL, POU3F3 immunoreactivity is present in the TAL, the MD and the CDs (nuclear staining pattern), and CD cells display a cytoplasmic staining pattern for the CD marker AQP2. Paraffin sections. Nuclear counterstain: Hemalum. Bars = 1 mm (left row), = 50 μm (middle and right row).

The abundance pattern of POU3F3 in different nephron segments and tubular compartments of homozygous *Pou3f3*^*L423P*^ mutant mice and non-mutant controls, were demonstrated by immunohistochemical detection of POU3F3, and co-localization with the TAL marker uromodulin (UMOD), and the CD marker aquaporin 2 (AQP2). Immunohistochemical detection of UMOD in the kidneys of non-mutant controls revealed a diffuse homogeneous cytoplasmic and distinct apical membrane staining pattern in epithelial cells of the TAL. In contrast, homozygous *Pou3f3*^*L423P*^ mutant mice of 12 days of age (data not shown) and of 60 weeks of age showed a less dense immunohistochemical UMOD staining pattern of individual TAL section profiles in the kidney, as compared to age and sex-matched heterozygous mutants and non-mutant control mice (Fig 6).

Immunohistochemical detection of POU3F3 revealed a nuclear staining pattern in epithelial cells of the TAL, the MD, and the CDs in homo- and heterozygous mutants and non-mutant control mice (Fig 6), as demonstrated by co-localization with the TAL marker UMOD and the CD marker AQP2. In contrast, POU3F3 staining was not detectable in glomerular cells. Simultaneous detection of UMOD and POU3F3 by double-immunohistochemistry proved the co-expression of both proteins in cells of the TAL (Fig 6). Likewise, the co-expression of POU3F3 and AQP2 in CD cells was shown by immunohistochemical detection of these proteins in consecutive sections of kidney tissue (Fig 6).

### Quantitative morphological findings

#### Quantitative morphological data of the renal cortex and the medulla

Corresponding to the significantly decreased kidney volumes, the total volumes of the renal cortex and of the medulla in 12-day-old and in 60-week-old homozygous *Pou3f3*^*L423P*^ mutant mice were consistently, on average one third, smaller than in age- and sex-matched heterozygous mutants and non-mutant control mice. In contrast, the total cortical and medullary volumes of heterozygous mutants and non-mutant control mice did not differ significantly (Tables 3 and 4). At 12 days of age, male and female mice of identical genotypes displayed approximately equal total volumes of the renal cortex and of the medulla (Table 3).

**Table 3:** Fractional and absolute volumes of cortex and medulla in the kidney of homozygous (HOM) and heterozygous (HET) *Pou3f3*^*L423P*^ mutant mice and control (CON) mice at 12 days of age.

Parameter	Sex	CON *(6/11)*		HET *(15/15)*		HOM *(7/5)*		Statistical significance*		
								*CON vs*. *HET*	*CON vs*. *HOM*	*HET vs*. *HOM*
V_V(Cortex/Kid)_	m	0.65	±0.05	0.66	±0.04	0.69	±0.04	*n*.*s*.	*n*.*s*.	*n*.*s*.
	f	0.65	±0.06	0.66	±0.04	0.71	±0.10	*n*.*s*.	*n*.*s*.	*n*.*s*.
V_(Cortex, Kid)_ [mm^3^]	m	42.9	±7.4	39.9	±8.4	29.5	±4.7	*n*.*s*.	*p<0*.*05*	*p<0*.*01*
	f	44.4	±10.1	40.1	±6.4	32.3	±7.2	*n*.*s*.	*p<0*.*05*	*n*.*s*.
V_V(Med/Kid)_	m	0.35	±0.05	0.34	±0.04	0.31	±0.04	*n*.*s*.	*n*.*s*.	*n*.*s*.
	f	0.35	±0.06	0.34	±0.04	0.29	±0.10	*n*.*s*.	*n*.*s*.	*n*.*s*.
V_(Med, Kid)_ [mm^3^]	m	22.6	±4.2	19.9	±3.1	13.4	±2.9	*n*.*s*.	*p<0*.*01*	*p<0*.*01*
	f	23.8	±5.7	20.8	±5.5	13.0	±4.8	*n*.*s*.	*p<0*.*01*	*n*.*s*.

Numbers of animals examined are given in brackets (male/female). Data are means ± SD.

*1-way ANOVA with Gabriel’s post hoc test; *n*.*s*. not significant. Male and female mice of the identical genotype did not display statistically significant differences in the listed parameters.

**Table 4:** Fractional and absolute volumes of cortex and medulla in the kidney of homozygous (HOM) and heterozygous (HET) *Pou3f3*^*L423P*^ mutant mice and control (CON) mice at 60 weeks of age.

Parameter	Sex	CON *(7/7)*		HET *(8/7)*		HOM *(6/6)*		Statistical significance*		
								*CON vs*. *HET*	*CON vs*. *HOM*	*HET vs*. *HOM*
V_V(Cortex/Kid)_	m	0.72 ^b^	±0.03	0.72	±0.04	0.71	±0.04	*n*.*s*.	*n*.*s*.	*n*.*s*.
	f	0.64 ^b^	±0.05	0.70	±0.05	0.75	±0.07	*n*.*s*.	*p<0*.*01*	*n*.*s*.
V_(Cortex, Kid)_ [mm^3^]	m	292.9 ^c^	±70.7	271.8 ^c^	±44.4	184.0 ^b^	±27.7	*n*.*s*.	*p<0*.*01*	*p<0*.*05*
	f	150.8 ^c^	±30.0	155.9 ^c^	±34.1	96.8 ^b^	±27.7	*n*.*s*.	*p<0*.*05*	*p<0*.*01*
V_V(Med/Kid)_	m	0.28 ^a^	±0.03	0.28	±0.04	0.29	±0.04	*n*.*s*.	*n*.*s*.	*n*.*s*.
	f	0.36 ^a^	±0.05	0.30	±0.05	0.25	±0.07	*n*.*s*.	*p<0*.*05*	*n*.*s*.
V_(Med, Kid)_ [mm^3^]	m	112.3	±25.6	106.3 ^b^	±25.6	77.4 ^b^	±24.6	*n*.*s*.	*n*.*s*.	*n*.*s*.
	f	86.3	±27.4	65.5 ^b^	±18.3	30.8 ^b^	±5.8	*n*.*s*.	*p<0*.*05*	*p<0*.*05*

Numbers of animals examined are given in brackets (male/female). Data are means ± SD.

*:1-way ANOVA with Gabriel’s post hoc test.

^a,b,c^: Statistically significant differences (a: p≤0.05, b: p≤0.01, c: p≤0.001) between male and female mice of the identical genotype; *n*.*s*. not significant.

At 60 weeks of age, however, the total cortical volumes of female animals were significantly smaller than in male homozygous (p<0.01) and heterozygous mutants (p<0.001). Correspondingly they also showed consistently significantly smaller total medullary volumes (p<0.01) than male mice (Table 4).

#### Quantitative morphological data of the TAL

**Fig 7:**
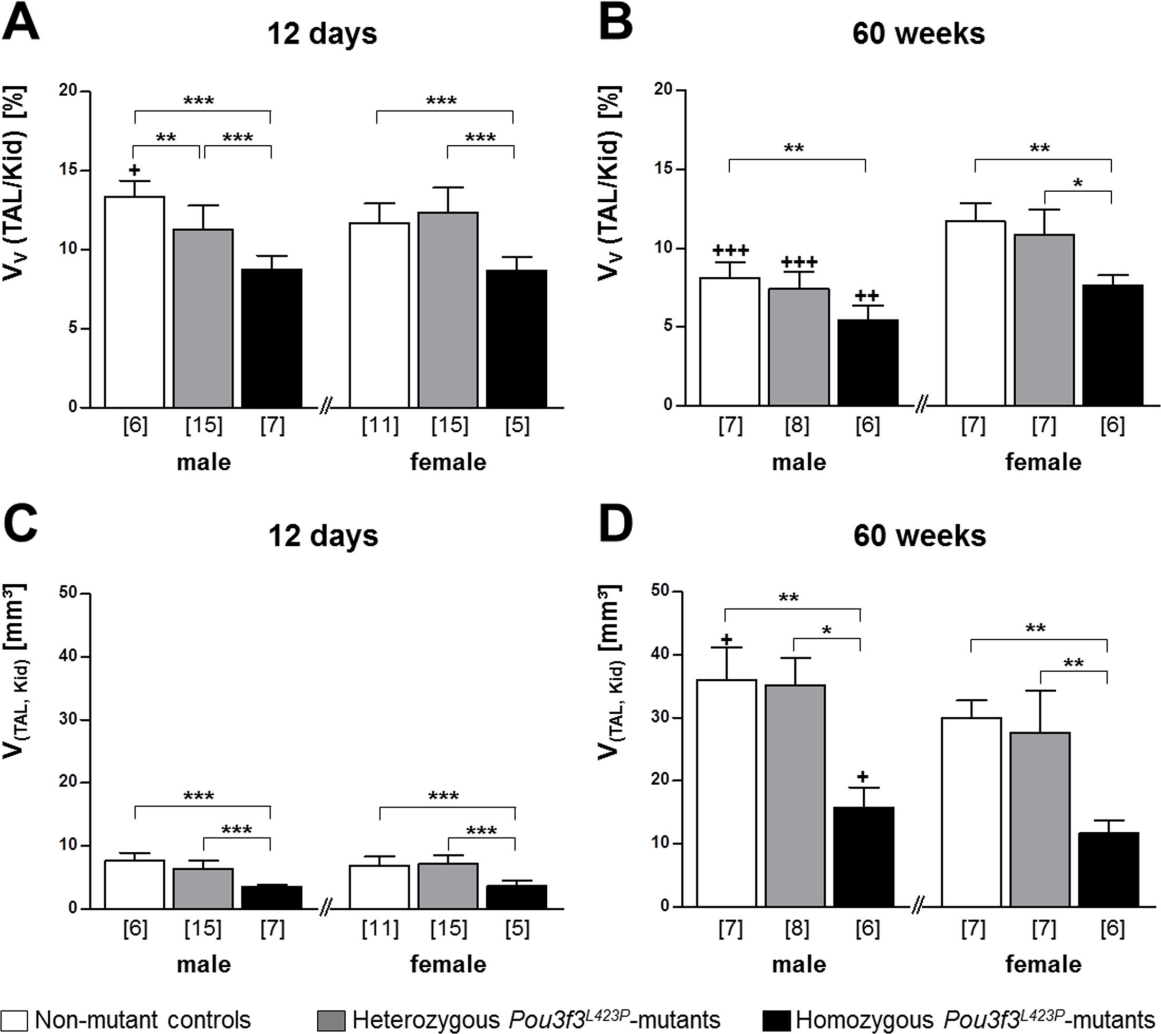
**Volume density of TAL cells in the kidney and absolute TAL volumes in 12-day-old (A, C) and 60-week-old (B, D) *Pou3f3***^***L423P***^ **mutant mice and non-mutant control mice.** The numbers of animals examined are given in parentheses. Data are means ± SD. Significant differences between homozygous and heterozygous mutants and non-mutant control mice are indicated by asterisks. *: p < 0.05, **: p < 0.01, ***: p < 0.001. Significant differences between male and female mice of identical genotypes are indicated by crosses. +: p < 0.05, ++: p < 0.01, +++: p < 0.001.

In juvenile and aged homozygous *Pou3f3*^*L423P*^ mutant mice, the total volume of the TAL of the loop of Henle (V_(TAL, Kid)_), as well as the volume density of the TAL in the kidney (V_V(TAL/Kid)_), were consistently significantly reduced (on average by 53% and 30% respectively), as compared to age- and sex-matched non-mutant control mice (p<0.001 at 12 days of age; p<0.01 at 60 weeks of age) (Fig 7). Compared to heterozygous mutants the V_(TAL, Kid)_ as well as the V_V(TAL/Kid)_ were also significantly smaller in 12-day-old homozygous mutants (p<0.001). In 60-week-old animals the V_(TAL, Kid)_ was significantly smaller in both male (p<0.05) and female (p<0.01) homozygous *Pou3f3*^*L423P*^ mutants, whereas the V_V(TAL/Kid)_ was only significantly reduced in female mice (p<0.05). In contrast, the total TAL volume and the volume density of the TAL in the kidney of heterozygous mutants and controls were not significantly different, except for the V_V(TAL/Kid)_ in male 12-day-old mice (p<0.001) (Fig 7).

#### Quantitative morphological data of the glomeruli

**Fig 8:**
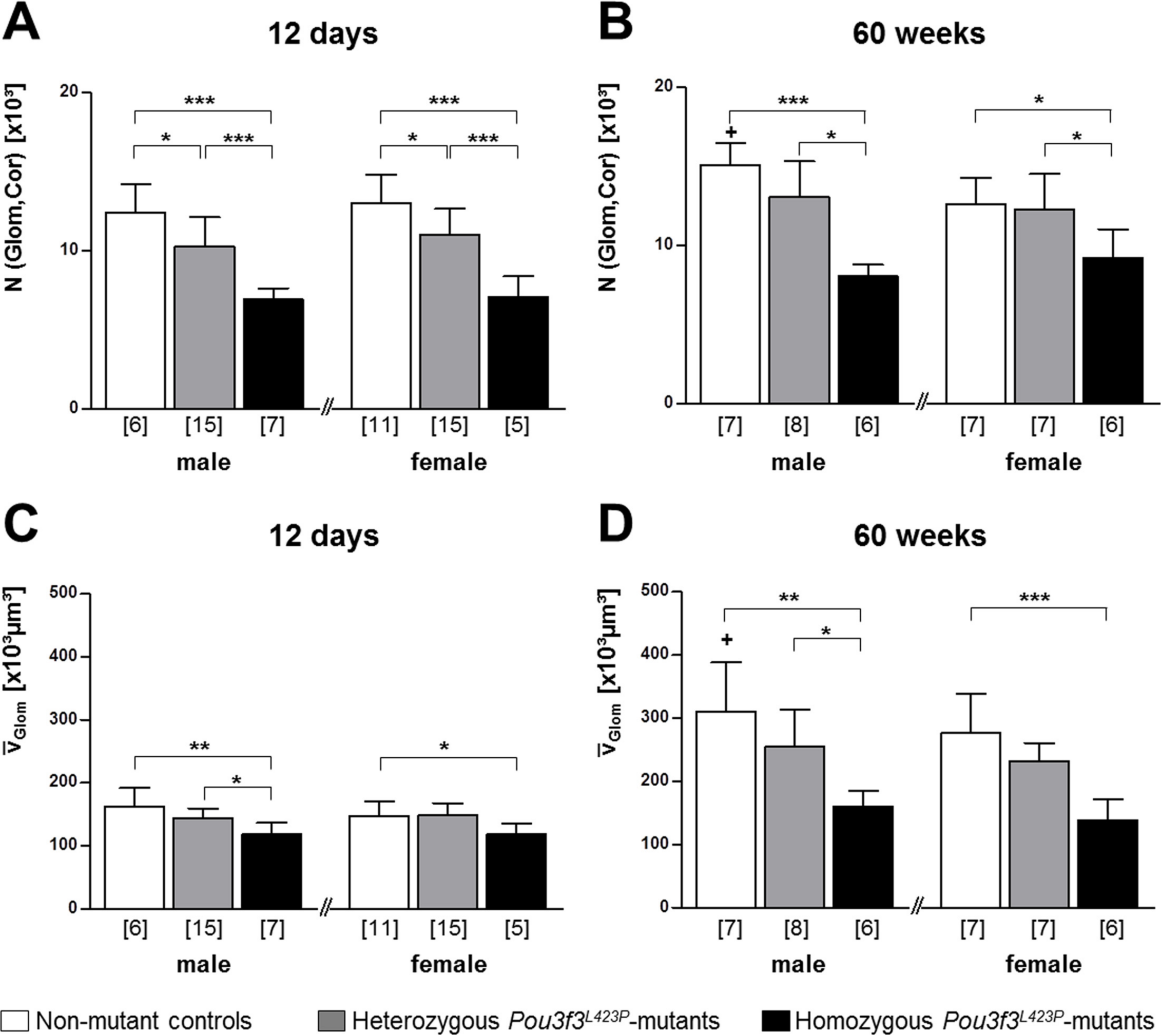
**Number of glomeruli per kidney and mean glomerular volumes in 12-day-old (A, C) and 60-week-old (B, D) *Pou3f3***^***L423P***^ **mutant mice and non-mutant control mice.** The numbers of examined animals are given in parentheses. Data are means ± SD. Significant differences between homozygous and heterozygous mutants and non-mutant control mice are indicated by asterisks. *: p < 0.05, **: p < 0.01, ***: p < 0.001. Significant differences between male and female mice of identical genotypes are indicated by crosses. +: p < 0.05, ++: p < 0.01, +++: p < 0.001.

For quantitative characterization of glomerular alterations, the volume density of the glomeruli in the kidney (V_V(Glom/Kid)_), the total volume (V_(Glom, Kid)_), and the number of nephrons (glomeruli) in the kidney (N_(Glom, Kid)_), and the mean glomerular volume (${\bar{v}}_{\left(\text{Glom}\right)}$) were determined in both 12-day-old, and in 60-week-old mutant mice and non-mutant controls (Tables 5 and 6; Fig 8). In 60-weeks-old *Pou3f3*^*L423P*^ mutant mice and non-mutant controls, additional quantitative parameters of glomerular morphology were determined, including the mean mesangium volume per glomerulus (V_(Mes, Glom)_), the mean capillary volume per glomerulus (V_(Cap, Glom)_), the average length of the capillaries per glomerulus (L_(Cap, Glom)_), the number of cells per glomerulus (N_(C, Glom)_), the mean podocyte volume (${\bar{\text{v}}}_{\left(\text{Pod}\right)}$), the filtration slit frequency (FSF), and the thickness of the glomerular basement membrane (GBM) (Tables 5–7; Fig 8).

**Table 5:** Quantitative stereological data of glomeruli in homozygous (HOM) and heterozygous (HET) Pou3f3^L423P^ mutant mice and control (CON) mice at 12 days of age.

Parameter	Sex	CON *(6/11)*		HET *(15/15)*		HOM *(7/5)*		Statistical significance*		
								*CON vs*. *HET*	*CON vs*. *HOM*	*HET vs*. *HOM*
V_V(Glom/Kid)_	m	0.04	±0.00	0.04	±0.01	0.04	±0.01	*n*.*s*.	*n*.*s*.	*n*.*s*.
	f	0.04	±0.01	0.04	±0.00	0.04	±0.01	*n*.*s*.	*n*.*s*.	*n*.*s*.
V_(Glom, Kid)_ [mm^3^]	m	1.68	±0.30	1.68	±0.37	1.23	±0.25	*n*.*s*.	*n*.*s*.	*p<0*.*05*
	f	1.85	±0.32	1.70	±0.37	1.33	±0.32	*n*.*s*.	*p<0*.*05*	*n*.*s*.
V_(Glom, Kid)_/body weight [10^3^ x mm^3^/g]	m	117	±15	117	±16	108	±18	*n*.*s*.	*n*.*s*.	*n*.*s*.
	f	131	±15	121	±22	110	±8	*n*.*s*.	*n*.*s*.	*n*.*s*.
N_V(Glom/Kid)_ [n/mm^3^]	m	390	±99	358	±66	306	±38	*n*.*s*.	*n*.*s*.	*n*.*s*.
	f	334	±78	317	±26	295	±33	*n*.*s*.	*p<0*.*05*	*n*.*s*.
N_(Glom,Kid)_ /BW [n/g]	m	176	±33	148	±40	122	±19	*n*.*s*.	*p<0*.*05*	*n*.*s*.
	f	189	±40	157	±20	120	±25	*p<0*.*05*	*p<0*.*001*	*p<0*.*05*
${\bar{\text{v}}}_{\left(\text{Glom}\right)}$/BW [10^3^ μm^3^/g]	m	22.6	±1.8	20.7	±4.1	20.8	±3.9	*n*.*s*.	*n*.*s*.	*n*.*s*.
	f	21.0	±2.6	21.2	±2.4	20.3	±4.0	*n*.*s*.	*n*.*s*.	*n*.*s*.

Numbers of examined animals are given in brackets (male/female). Data are means ± SD.

*:1-way ANOVA with Gabriel’s post hoc test; *n*.*s*. not significant. Male and female mice of the identical genotype did not display statistically significant differences in the listed parameters.

**Table 6:** Quantitative stereological data of glomeruli and glomerular subcompartments in homozygous (HOM) and heterozygous (HET) *Pou3f3*^*L423P*^ mutant mice and control (CON) mice at 60 weeks of age.

Parameter	Sex	CON *(7/7)*		HET *(8/7)*		HOM *(6/6)*		Statistical significance*		
								*CON vs*. *HET*	*CON vs*. *HOM*	*HET vs*. *HOM*
V_V(Glom/Kid)_	m	0.018 ^c^	±0.004	0.017 ^c^	±0.003	0.013 ^c^	±0.002	*n*.*s*.	*p<0*.*05*	*p<0*.*05*
	f	0.030 ^c^	±0.003	0.026 ^c^	±0.005	0.023 ^c^	±0.004	*n*.*s*.	*p<0*.*05*	*n*.*s*.
V_(Glom, Kid)_ [mm^3^]	m	5.35	±1.57	4.69	±0.97	2.28	±0.59	*n*.*s*.	*p<0*.*01*	*p<0*.*01*
	f	4.44	±0.89	4.00	±0.81	2.27	±0.82	*n*.*s*.	*p<0*.*001*	*p<0*.*01*
V_(Glom, Kid)_/body weight [10^3^ x mm^3^/g]	m	136	±41	121	±21	85	±17	*n*.*s*.	*p<0*.*05*	*n*.*s*.
	f	120	±27	136	±61	98	±28	*n*.*s*.	*n*.*s*.	*n*.*s*.
N_V(Glom/ Kid)_ [n/mm^3^]	m	72 ^b^	±20	62 ^b^	±10	59 ^b^	±9	*n*.*s*.	*n*.*s*.	*n*.*s*.
	f	114 ^b^	±27	110 ^b^	±38	130 ^b^	±20	*n*.*s*.	*n*.*s*.	*n*.*s*.
N_(Glom, Kid)_ /BW [n/g]	m	386	±43	341	±70	301 ^a^	±38	*n*.*s*.	*p<0*.*05*	*n*.*s*.
	f	344	±85	409	±131	403 ^a^	±75	*n*.*s*.	*n*.*s*.	*n*.*s*.
${\bar{\text{v}}}_{\left(\text{Glom}\right)}$/BW [10^3^ μm^3^/g]	m	7.9	±1.9	6.6	±1.2	6.0	±0.7	*n*.*s*.	*p<0*.*05*	*n*.*s*.
	f	6.9	±0.9	7.9	±2.8	6.1	±1.2	*n*.*s*.	*n*.*s*.	*n*.*s*.
V_V(Mes/Glom)_	m	0.28	±0.04	0.23	±0.02	0.26	±0.08	*n*.*s*.	*n*.*s*.	*n*.*s*.
	f	0.25	±0.04	0.25	±0.04	0.26	±0.07	*n*.*s*.	*n*.*s*.	*n*.*s*.
V_(Mes, Glom)_ [x10^3^ μm^3^]	m	86 ^a^	±18	58	±12	40	±8	*p<0*.*01*	*p<0*.*001*	*n*.*s*.
	f	66 ^a^	±9	57	±11	35	±6	*n*.*s*.	*p<0*.*01*	*p<0*.*05*
V_V(Cap/Glom)_	m	0.44^b^	±0.03	0.51	±0.02	0.47	±0.05	*p<0*.*01*	*n*.*s*.	*n*.*s*.
	f	0.50 ^b^	±0.04	0.50	±0.06	0.44	±0.06	*n*.*s*.	*n*.*s*.	*n*.*s*.
V_(Cap, Glom)_ [x10^3^ μm^3^]	m	137	±42	130	±30	76	±18	*n*.*s*.	*p<0*.*05*	*p<0*.*05*
	f	139	±37	114	±15	63	±21	*n*.*s*.	*p<0*.*01*	*n*.*s*.
V_V(Pod/Glom)_	m	0.28	±0.02	0.26	±0.01	0.27	±0.04	*n*.*s*.	*n*.*s*.	*n*.*s*.
	f	0.26	±0.04	0.26	±0.04	0.29	±0.03	*n*.*s*.	*n*.*s*.	*n*.*s*.
${\bar{\text{v}}}_{\left(\text{Pod},\;\text{Glom}\right)}$ [x10^3^ μm^3^]	m	87	±23	67	±18	44	±12	*n*.*s*.	*p<0*.*01*	*n*.*s*.
	f	71	±22	60	±15	41	±12	*n*.*s*.	*p<0*.*05*	*n*.*s*.
L_V(Cap/Glom)_ [mm/mm^3^]	m	0.014	±0.003	0.018	±0.003	0.019	±0.004	*n*.*s*.	*n*.*s*.	*n*.*s*.
	f	0.017	±0.003	0.020	±0.003	0.020	±0.004	*n*.*s*.	*n*.*s*.	*n*.*s*.
L_(Cap, Glom)_ [mm]	m	4.2	±0.5	4.3	±0.4	3.0	±0.4	*n*.*s*.	*p<0*.*001*	*p<0*.*001*
	f	4.5	±0.6	4.7	±0.5	2.8	±0.5	*n*.*s*.	*p<0*.*05*	*p<0*.*05*
L_(Glom-cap, Kid)_ [m]	m	72	±12	81	±14	42	±9	*n*.*s*.	*p<0*.*05*	*p<0*.*01*
	f	76	±13	81	±16	45	±11	*n*.*s*.	*p<0*.*05*	*p<0*.*01*
N_V(C/Glom)_ [n/10^5^ μm^3^]	m	95	±12	101	±11	105 ^a^	±7	*n*.*s*.	*n*.*s*.	*n*.*s*.
	f	108	±16	111	±6	128 ^a^	±16	*n*.*s*.	*n*.*s*.	*n*.*s*.
N_(C, Glom)_	m	277	±45	258	±66	168	±20	*n*.*s*.	*p<0*.*01*	*p<0*.*05*
	f	295	±65	252	±24	175	±36	*n*.*s*.	*p<0*.*01*	*n*.*s*.
N_V(M-E/Glom)_ [n/10^5^ μm^3^]	m	58	±8	61	±14	57 ^a^	±4	*n*.*s*.	*n*.*s*.	*n*.*s*.
	f	68	±13	66	±2	74 ^a^	±16	*n*.*s*.	*n*.*s*.	*n*.*s*.
N_(M-E,Glom)_	m	173	±43	159	±63	91	±16	*n*.*s*.	*p<0*.*05*	*n*.*s*.
	f	189	±70	153	±22	104	±36	*n*.*s*.	*p<0*.*01*	*n*.*s*.
N_V (Pod/Glom)_ [n/10^5^ μm^3^]	m	37	±8	41	±8	48	±6	*n*.*s*.	*n*.*s*.	*n*.*s*.
	f	40	±10	44	±6	54	±13	*n*.*s*.	*n*.*s*.	*n*.*s*.
N_(Pod,Glom)_	m	104	±5	100	±9	76	±5	*n*.*s*.	*p<0*.*001*	*p<0*.*01*
	f	106	±7	100	±3	72	±7	*n*.*s*.	*p<0*.*001*	*p<0*.*01*
${\bar{\text{v}}}_{\left(\text{Pod}\right)}$	m	811	±204	675	±161	574	±122	*n*.*s*.	*n*.*s*.	*n*.*s*.
	f	602	±141	594	±161	572	±165	*n*.*s*.	*n*.*s*.	*n*.*s*.

Numbers of animals examined are given in brackets (male/female). Data are means ± SD.

*:1-way ANOVA with Gabriel’s post hoc test.

^a,b,c^: Statistically significant differences (a: p≤0.05, b: p≤0.01, c: p≤0.001) between male and female mice of the identical genotype; *n*.*s*. not significant.

**Table 7:** True harmonic mean thickness of the glomerular basement membrane (GBM) and filtration slit frequency (FSF) in homozygous (HOM) and heterozygous (HET) *Pou3f3*^*L423P*^ mutant mice and control (CON) mice at 60 weeks of age.

Parameter	Sex	CON *(7/7)*		HET *(8/7)*		HOM *(6/6)*		Statistical significance*		
								*CON vs*. *HET*	*CON vs*. *HOM*	*HET vs*. *HOM*
Th_(GBM)_ [nm]	m	178	±12	185	±11	156	±13	*n*.*s*.	*n*.*s*.	*p<0*.*05*
	f	164	±6	178	±12	146	±4	*n*.*s*.	*n*.*s*.	*p<0*.*05*
FSF [n/mm GBM]	m	2504	±194	2568	±179	2573	±67	*n*.*s*.	*n*.*s*.	*n*.*s*.
	f	2560	±189	2517	±61	2631	±158	*n*.*s*.	*n*.*s*.	*n*.*s*.

Numbers of animals examined are given in brackets (male/female). Data are means ± SD.

*:1-way ANOVA with Gabriel’s post hoc test.

^a,b,c^: Statistically significant differences (a: p≤0.05, b: p≤0.01, c: p≤0.001) between male and female mice of the identical genotype; *n*.*s*. not significant.

#### Volume density of the glomeruli in the kidney, total and relative glomerular volume

At 12 days of age, the volume densities of the glomeruli in the kidney were not significantly different between homo- and heterozygous mutants and control mice (Table 5). The total volume of the glomeruli, however, was consistently smaller in homozygous mutants *vs*. control mice, as well as in homo- *vs*. heterozygous mutants, with statistically significant differences in female homozygous mutants *vs*. controls and in male homo- *vs*. heterozygous mutants (p<0.05). The total glomerular volumes of heterozygous mutants and control mice were not significantly different (Table 5).

The relative total glomerular volume (total glomerular volume in the kidney in relation to body weight, V_(Glom, Kid)_/BW) was not significantly different between 12-day-old homo- and heterozygous *Pou3f3*^*L423P*^ mutant mice and non-mutant controls of both sexes (Table 5).

At 60 weeks of age, however, both the volume densities of the glomeruli in the kidney (p<0.05) and the total volume of the glomeruli (male p<0.01, female p<0.001) in homozygous mutant mice were significantly lower as compared to non-mutant control mice. Except for the glomerular volume density in the kidneys of female mice, the total glomerular volumes (p<0.01) and glomerular volume densities (p<0.05) in the kidney of homozygous mutants were also significantly reduced as compared to heterozygous mutant mice. In contrast, V_V(Glom/Kid)_ and V_(Glom, Kid)_ were not statistically significantly different between heterozygous *Pou3f3*^*L423P*^ mutant mice and non-mutant controls (Table 6). In contrast to 12-day-old mice, the relative total glomerular volumes in 60-week-old homozygous *Pou3f3*^*L423P*^ mutants were markedly reduced, as compared to heterozygous mutant mice and non-mutant control mice, with a statistically significant difference in male homozygous mutants *vs*. controls (p<0.05). In contrast, the relative total glomerular volumes of 60-week-old heterozygous mutants and control mice were not significantly different (Table 6).

#### Total and relative nephron number, mean glomerular volume, and relative mean glomerular volume

Quantitative stereological analyses revealed a marked reduction of the numbers of nephrons (glomeruli) in juvenile and aged homozygous *Pou3f3*^*L423P*^ mutants as compared to heterozygous mutants and non-mutant controls, being highly significant in both sexes at 12 days of age (p<0.001). At 60 weeks of age, nephron numbers were also significantly lower in homozygous mutants as compared to heterozygous (p<0.05) and non-mutant controls (male p<0.001, female p<0.05). On average, male and female homozygous mutants showed 40% lower nephron numbers than age and sex-matched, non-mutant controls. Whereas at 12 days of age, heterozygous mutants displayed also significantly lower nephron numbers than non-mutant controls (p<0.05), the nephron numbers in 60-week-old heterozygous mutants and controls did not differ significantly (Fig 8). There were no statistically significant differences between the number of glomeruli in 12-day-old mice and 60-week-old mice of identical genotype and gender.

In addition to the significantly lower total number of nephrons, the relative nephron numbers (total nephron number per body weight, N_(Glom,Kid)_/BW) were reduced, reaching statistical significance in 12-day-old male (p<0.05) and female (p<0.001) homozygous mutants, as well as in male homozygous mutants of 60 weeks of age (p<0.05) compared to non-mutant control mice (Tables 5 and 6). The relative nephron numbers of homozygous *vs*. heterozygous mutants and of heterozygous mutants *vs*. control mice were only significantly reduced in female mice of 12 days of age (p<0.05), but not in 12-day-old male mice, or in mice of 60 weeks of age (Tables 5 and 6).

Confirming the qualitative histological findings, the stereologically determined mean glomerular volumes of homozygous *Pou3f3*^*L423P*^ mutants were significantly reduced as compared to control mice in both juvenile (male p<0.01, female p<0.05) and aged animals (male p<0.01, female p<0.001) (Fig 8). On average, male and female homozygous mutants of both examined age groups displayed 27% smaller mean glomerular volumes than their age- and sex-matched controls. In both examined age groups, a statistically significant difference between the lower mean glomerular volumes in homo- *vs*. heterozygous mutant mice was only present in male mice (p<0.05). The mean glomerular volumes in heterozygous mutants and in control mice were not significantly different between both examined stages of age (Fig 8). In contrast to the striking reduction of the mean glomerular volumes, the relative mean glomerular volumes (related to body weight) did not display statistically significant differences between homo- and heterozygous mutants and non-mutant control mice of 12 days or 60 weeks of age (Tables 5 and 6).

#### Additional quantitative parameters of glomerular morphology of 60-week-old mice

In 60-week-old mice, the volume densities of the mesangium in the glomeruli were similar in all examined genotype groups. Corresponding to their lower mean glomerular volume, homozygous *Pou3f3*^*L423P*^ mutant mice also displayed lower mean mesangial volumes per glomerulus (V_(Mes, Glom)_) than heterozygous mutants and control mice, and heterozygous mutants displayed lower mean mesangial volumes per glomerulus than control mice (Table 6). These differences reached statistical significance in homozygous *vs*. control mice (male p<0.001, female p<0.01), female homozygous *vs*. heterozygous mutant mice (p<0.05), and male heterozygous mutants *vs*. control mice (p<0.01).

The mean capillary volume per glomerulus (V_(Cap, Glom)_) of homozygous *Pou3f3*^*L423P*^ mutant mice of both sexes was significantly lower as compared to non-mutant controls (p<0.05). Compared to heterozygous mutants only male homozygous mutants showed a statistical significant reduction (p<0.05). The mean length of the capillaries per glomerulus (L_(Cap, Glom)_) was significantly reduced in homozygous mutants as compared to both heterozygous mutants and non-mutant controls (male p<0.001, female 0.05). Concordingly the total length of the glomerular capillaries in the kidney (L_(Glom-cap, Kid)_) in homozygous *Pou3f3*^*L423P*^ mutant mice was consistently significantly lower than in heterozygous mutants (p<0.01) and non-mutant control mice (p<0.05). In contrast, these parameters were not significantly different between heterozygous mutant mice and controls (Table 6).

Corresponding to their smaller mean glomerular volumes, male and female homozygous *Pou3f3*^*L423P*^ mutant mice exhibited significantly lower numbers of cells per glomerulus than sex-matched control mice (p<0.01, on average -37%). Compared to sex-matched heterozygous mutant mice, male, but not female homozygous mutant mice also displayed a significantly lower total number of cells per glomerulus (p<0.05). In contrast, the total number of cells per glomerulus was not significantly different between heterozygous mutants and control mice of both sexes (Table 6). Concordantly, homozygous *Pou3f3*^*L423P*^ mutant mice exhibited significantly reduced numbers of podocytes (p<0.001) and of mesangial and endothelial cells per glomerulus as compared to control mice (male p<0.05, female p<0.01). Except for the significantly reduced number of podocytes per glomerulus in male homo- *vs*. heterozygous mutants (p<0.01), heterozygous mutant mice did not display significantly different podocyte or mesangial and endothelial cell numbers per glomerulus as compared to homozygous mutants or non-mutant control mice (Table 6). The mean podocyte volumes were not significantly different between mice of the three examined genotypes (Table 6). The true harmonic mean thickness of the GBM of homozygous *Pou3f3*^*L423P*^ mutant mice of both sexes was significantly smaller (about -20%) than in heterozygous mutants (p<0.05), whereas there were no significant differences in GBM thickness between homozygous mutants and controls, or between heterozygous mutants and control mice (Table 7). The number of filtration slits between adjacent podocyte foot processes per length of the GBM was virtually equal in all examined groups of mice (Table 7).

### Renal POU3F3 mRNA and protein abundance

**Fig 9:**
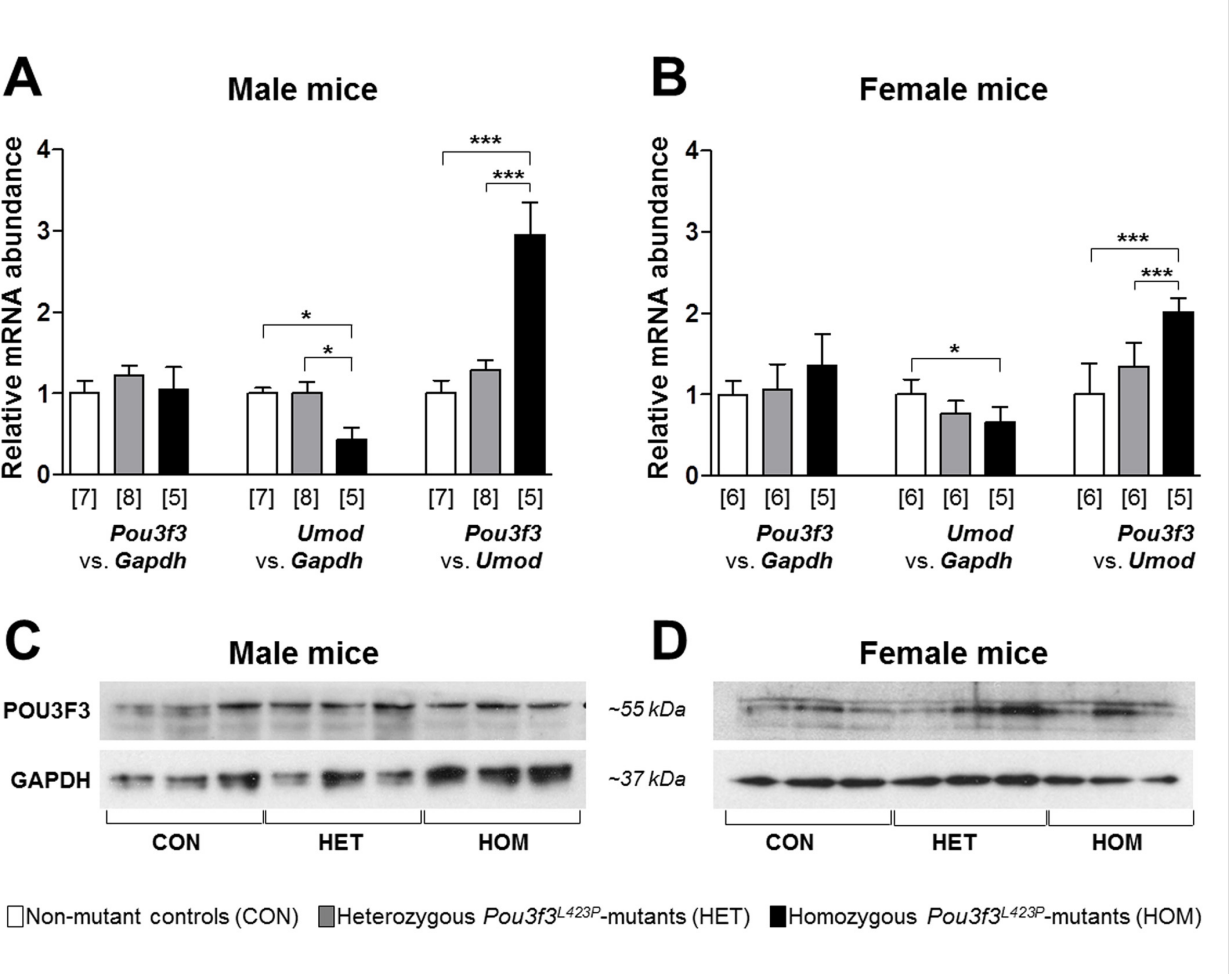
**RT-qPCR (A, B) and Western blot analyses (C, D) of relative POU3F3 abundances in the kidneys of 60-week-old (B, D) homozygous (HOM) and heterozygous (HET) *Pou3f3***^***L423P***^ **mutant mice and non-mutant control mice (CON).** The relative mRNA abundances (*Pou3f3* vs. *Gapdh*, *Umod* vs. *Gapdh*, and *Pou3f3* vs. *Umod*) were normalized to the corresponding mean relative mRNA abundances of non-mutant control mice. The numbers of animals examined are given in parentheses. Data are means ± SD. Significant differences between homozygous and heterozygous mutants and non-mutant control mice are indicated by asterisks. *: p < 0.05, **: p < 0.01, ***: p < 0.001.

Quantitative RT-real time PCR (RT-qPCR) analyses (Fig 9A and 9B) did not reveal statistically significantly different relative renal *Pou3f3* mRNA abundances (relative to *Gapdh*) in homozygous and heterozygous *Pou3f3*^*L423P*^ mutant mice and non-mutant control mice. The relative renal mRNA abundances of *Umod* (relative to *Gapdh*), however, were significantly lower in homozygous *Pou3f3*^*L423P*^ mutant mice than in non-mutant control mice (p<0.05), and in male homo- *vs*. heterozygous mutants (p<0.05). Correspondingly, homozygous *Pou3f3*^*L423P*^ mutant mice displayed significantly higher relative renal *Pou3f3* mRNA abundances (relative to *Umod*) than heterozygous mutants (p<0.001) and non-mutant control mice (p<0.001). By Western-blot analysis, renal POU3F3 protein was detectable in total kidney protein lysates of homozygous and heterozygous *Pou3f3*^*L423P*^ mutant mice, as well as in non-mutant control mice (Fig 9C and 9D).

## Discussion

In the mammalian kidney, development of functioning nephrons in adequate numbers (*i*.*e*., nephron endowment) depends on the proper induction of nephrons in the embryonic metanephros, the subsequent transition of the renal vesicles into segmented nephrons (nephron patterning), and the maturation of the different nephron segments. These processes are controlled by complex, temporally and spatially coordinated interaction networks of several different transcription- and growth factors, receptors, and signaling molecules/cascades that regulate nephro-nephronogenesis by activation or repression of genes in specific cell types [4,6,7]. The early fetal induction of ureteric bud branching, the accurate signaling between the tips of the developing ureteric bud and the surrounding metanephric mesenchyme leading to formation the renal vesicle (nephron induction), the subsequent metanephric mesenchymal to epithelial transformation in the renal vesicle and its conversion to primitive nephrons (comma- and S-shaped bodies) is controlled by transcriptional pathways, *inter alia* involving the glial-derived neurotrophic factor (GDNF) and CRET (ret proto-oncogene) signaling pathway, the WNT-beta-catenin cascade, and transcription- and inducing factors as EYA1 (eyes absent homolog 1 transcriptional coactivator and phosphatase 1), LIF (leukemia inhibitory factor), FGF2 (fibroblast growth factor 2), and TGFB2 (transforming growth factor beta 2) [4,6,7]. The subsequent patterning of the nephron, *i*.*e*., the formation of distinct differentiated tubular segments along the proximal–distal nephron axis, and the invasion of capillaries and further differentiation to immature glomeruli is governed by transcription factors such as WT1 (Wilms’ tumor 1), LHX1 (LIM homeobox 1), POU3F3, and DLL1 (delta-like 1). Here, Notch–Delta pathway signaling particularly determines the patterning of proximal tubular nephron segments, whereas the fate of the distal nephron is specified by POU3F3 and IRX (iroquois homeobox) transcription factors [4,6,7]. However, the exact functions, the manifold interactions, and the regulatory hierarchies of all different factors involved in nephro-nephronogenesis are not yet fully clarified. In mice, the final maturation of the glomeruli, different tubular nephron segments, and the CDs is completed around postnatal day 7–10 [42].

The putative functions of several nephrogenetic factors were discovered and studied in genetically modified rodent models, where the complete or partial loss of function of a single growth or transcription factor led to a distinct phenotype, as, *e*.*g*., a typical malformation of the entire kidney, or of a distinct nephron segment [43–45]. Likewise, the important role of POU3F3 in nephron patterning during nephrogenesis, particularly for formation, differentiation, elongation, and maturation of the thick ascending limb (TAL) of the loop of Henle and the differentiation of the macula densa (MD) was discovered in *Pou3f3* knock-out mice and reported by Nakai *et al*. in 2003 [5]. In the developing nephron, *Pou3f3* expression was detected in distal renal vesicles, S-shaped bodies, and nephron regions that develop into the TAL, the MD, and the distal convoluted tubule, where *Pou3f3* expression also persists in the mature nephron. In the kidneys of homozygous *Pou3f3* knock-out mice, the TAL is largely absent at birth [5]. New-born homozygous *Pou3f3* knock-out mice exhibit increased plasma urea and potassium levels and die from renal failure within 24 hours after birth [5].

Possible mechanisms how POU3F3 deficiency triggers underdevelopment of the TAL include reduced cell proliferation and early onset of apoptosis in the primitive nephron loops of homozygous *Pou3f3* knock-out embryos [5].

Indicating a gene-dosage dependent role of POU3F3 in regulation of gene expression in the TAL, adult heterozygous *Pou3f3* knock-out mice display reduced expression levels of genes normally expressed in the TAL (including *Umod*, *Nkcc2*, and different K^+^ and Cl^-^ channels). However, there is no evidence for an altered cellular morphology or limitation of normal TAL function heterozygous knock-out mice, as shown by histological and ultrastructural analyses and by unaltered blood/serum levels of urea, creatinine, Na^+^, K^+^, Cl^-^, and normal urine osmolality and volumes [5].

The present study analyzed *Pou3f3*^*L423P*^-mice, a mutant mouse line that was recently generated in the Munich ENU-Mouse-Mutagenesis project [15,16]. The point mutation in the *Pou3f3* gene is not lethal and, in contrast to homozygous *Pou3f3*-knock-out mice, homozygous *Pou3f3*^*L423P*^ mutant mice are viable and fertile [16] and thus can be used to study the effects of the POU3F3 mutation on renal development, morphology, and function in the postnatal life. Adult homozygous *Pou3f3*^*L423P*^ mutant mice have previously been shown to display increased plasma urea levels, along with several other metabolic changes, including increased creatinine, chloride, and potassium serum levels, as well as a decreased bone mineral density and bone mineral content, indicative for impaired renal function, [5,16].

To analyze both immediate developmental effects on the nephronogenesis as well as long-term consequences of the *Pou3f3*^*L423P*^ mutation on renal function and morphology, homo- and heterozygous mutant mice and non-mutant controls were examined at 12 days, around completion of nephronogenesis [12], and at 60 weeks of age. For a comprehensive morphological analysis of the development, growth and cellular composition of distinct nephron segments, such as the TAL and the glomeruli, qualitative histological and ultrastructural examinations, as well as unbiased, design-based quantitative stereological methods were applied [46–48]. The results of these analyses confirmed the significant role of POU3F3 in nephrogenesis, and particularly in the development of the TAL, which is in line with the findings in *Pou3f3* knock-out mice reported by Nakai *et al*. [5]. Both 12-day-old and 60-week-old homozygous *Pou3f3*^*L423P*^ mutant mice displayed significantly smaller kidney weights and volumes than non-mutant controls, as well as significantly lower total TAL volumes and volume densities of the TAL in the kidney. Given the important function of the TAL in renal nitrogen metabolism [8,49], the incomplete formation of the TAL might provide an explanation for the increased serum urea concentrations detected in homozygous *Pou3f3*^*L423P*^ mutants [5,16].

In further accordance with the findings by Nakai *et al*., the present study immunohistochemically detected POU3F3 in the TAL and the MD of both juvenile and aged, mutant and non-mutant mice, confirming the importance of POU3F3 in fetal development of the TAL, and providing additional evidence for its putative role in maintenance of the function of the TAL in the developed kidney [5]. Additionally, the present study detected a positive POU3F3 immunostaining in CD cell nuclei of the mature kidney, which has not been described in the developing kidney [5].

Basically, a meaningful quantitative comparison of the effects of a complete POU3F3 deficiency in *Pou3f3* knock-out mice with the consequences of the *Pou3f3*^*L423P*^ mutation on the extent of TAL under-development is not possible, since mice of different ages and different genetic backgrounds were investigated, and since the extent of TAL under-development was only semiquantitatively assessed in the study of Nakai *et al*. [5]. However, marked alterations of the cellular morphology and the ultrastructure of TAL cells, as observed in *Pou3f3* knock-out mice [5], were not present in homozygous *Pou3f3*^*L423P*^ mutants examined in the present study. In addition to the underdevelopment of the TAL, quantitative stereological analyses revealed a striking, significant reduction of the absolute nephron numbers and the relative numbers of nephrons per body weight, as well as a significant decrease of the mean glomerular volume (but not of the relative mean glomerular volume per body weight) in the kidneys of homozygous *Pou3f3*^*L423P*^ mutants, present at 12 days of age. The same findings were also present in aged, 60-week-old homozygous *Pou3f3*^*L423P*^ mutant mice, except for the relative nephron number, which was only significantly reduced in male, but not in female homozygous mutants *vs*. control mice. In *Pou3f3* knock-out mice, a comparable effect of abolished POU3F3 function on reduction of the numbers and sizes of glomeruli has not been reported, probably because nephronogenesis and glomerular development are still in progress in newborn mice [12], or due to application of a less accurate method for quantification and sizing of glomeruli. Therefore, in addition to its known role in nephron patterning (*i*.*e*., development of the TAL), the results of the present study show that POU3F3 is also involved in determination of the final nephron number (nephron endowment) of the mature mouse kidney. In this context, the reduction of the absolute nephron number in homozygous mutants vs. control mice actually appears to represent the decisive parameter, rather than alterations of the relative nephron number. Studies in different mouse strains did reveal a correlation between kidney- and body weight at birth and at adulthood, but not between the number of nephrons and body weight or between nephron number and kidney size [50]. Therefore, the finding that a significant reduction of the relative nephron number, as observed in male and female 12-day-old homozygous *Pou3f3*^*L423P*^ mutants, was only present in male, but not in female homozygous mutants of 60 weeks of age does not contradict a role of POU3F3 for nephron endowment of the kidney.

Long-term consequences of the quantitative reduction of the TAL were essentially restricted to functional alterations of the tubular system, like alterations in serum and urine parameters (i.e., elevation of serum urea concentrations and decreased specific urine gravities), whereas no further pathomorphological alterations of the tubular system were detected in the kidneys of aged homozygous *Pou3f3*^*L423P*^ mutants. In these mice, the *Pou3f3* mutation and the altered TAL did also not exert detectable effects on glomerular function, as the serum cystatin C concentration, urine albumin excretion, and blood pressure were unaffected in aged mutant mice. Apart from the reduced nephron number and the diminished size of the glomeruli, no functional, qualitative or quantitative morphological alterations were detected in aged, 60-week-old *Pou3f3*^*L423P*^ mutant mice. Protein analyses of spot urine samples did not reveal any evidence for an increased urinary albumin excretion in *Pou3f3*^*L423P*^ mutant mice as compared to non-mutant controls. These findings are in line with previously reported results of analyses of 24-hour urine samples, where the urinary albumin and total protein excretion per day was not significantly different between homozygous *Pou3f3*^*L423P*^ mutants and control mice of 15 weeks of age [16]. Moreover, no evidence of histological or ultrastructural glomerular lesions was present in *Pou3f3*^*L423P*^ mutants, and quantitative morphological parameters, such as the GBM thickness, or the mean podocyte volume, representing sensitive and early indicators of glomerular damage [17,27,32] were similar in mutant mice and non-mutant controls. Thus, the reduced nephron endowment in mutant mice did obviously not cause functionally relevant alterations of the glomerular morphology.

In the present study, RT-qPCR and Western-blot analyses did not reveal evidence for a reduced mRNA or protein abundance of POU3f3 in the kidneys of homozygous or heterozygous *Pou3f3*^*L423P*^ mutant mice, as compared to non-mutant control mice. A possible explanation for the observed differences in *Pou3f3* knock-out and *Pou3f3*^*L423P*^ mutant mice, such as the neonatal death in *Pou3f3* knock-out mice, might lie in an altered, but not completely abolished activity of the mutant POU3F3 protein. The *Pou3f3*^*L423P*^ mutation is located in the conserved homeobox domain of the protein and might therefore alter the transcriptional regulation of all or a subset of POU3F3 target genes. Additionally, lack of distinct POU3F3 functions in homozygous mutant mice might partially be compensated by unknown other, synergistically acting factors. Finally, the milder phenotype of heterozygous *Pou3f3* knock-out mice and of heterozygous *Pou3f3*^*L423P*^ mutants can also be interpreted as a gene-dose dependent effect [5]. However, further studies are necessary to clear the exact molecular mechanisms, how POU3F3 participates in regulation of nephrogenesis and maintenance of TAL function in the mature kidney, including the timely and spatially coordinated transcriptional control of its target-genes, interaction with other factors, and potential modulation of different signaling pathways regulating cell proliferation, differentiation, and apoptosis [5]. In these studies, *Pou3f3*^*L423P*^ mutant mice might prove a useful model system.

Finally, the observed reduction in the nephron number in homozygous *Pou3f3*^*L423P*^ mutant mice is of particular interest, since a low nephron endowment is considered as an important individual risk factor for development of diverse chronic kidney diseases in humans [51–53]. Several studies in human beings and animal models have shown that low nephron numbers are often associated with glomerular hypertrophy [43,54,55], a pathogenetic key lesion for development of glomerulosclerosis [56,57], and with hypertension [52,58–60]. In this context, the *a priori* low number of nephrons is seen as a “first hit”, predisposing the kidney to a facilitated development of glomerular hypertrophy, promoting subsequent establishment of glomerulosclerotic lesions, progressive loss of nephrons and renal function, and establishment of hypertension, if challenged by a “second hit”, as e.g., a primary nephropathy, diabetes mellitus, or obesity [53,61–63]. The exact pathomechanisms how a low nephron endowment may favor the development of glomerular hypertrophy (e.g., by induction of compensatory glomerular growth [43] mediated by glomerular growth factors [56,64]) or hypertension (e.g., by inappropriate activation of the renin angiotensin system, impaired tubular salt excretion causing salt and volume retention, or by structural alterations of peritubular vessels) are, however, not completely understood yet [43]. There are currently only few murine models of genetically determined low nephron numbers, such as heterozygous *Gdnf* knock-out mice [43,65]. Compared to non-mutant controls, these mice display approximately 33% lower nephron numbers, while their body- and kidney weights are not altered [43,65]. However, apart from a higher occurrence of unilateral renal agenesis in heterozygous *Gdnf* knock-out mice, the reduction of nephron numbers also leads to development of a specific pattern of glomerular alterations, such as glomerular hypertrophy with hyperplasia of endothelial and mesangial glomerular cells and thickening of the glomerular basement membrane (GBM), predisposing the mice to development of glomerular sclerosis [43]. In contrast, a comparable reduction of nephron numbers in homozygous *Pou3f3*^*L423P*^ mutant mice by 37% on the average was not associated with an “own” pattern of pathological alterations of glomerular morphology and function, such as glomerular hypertrophy, glomerular mesangial and endothelial cell hyperplasia, thickening of the GBM, or albuminuria. Correspondingly, the aged *Pou3f3*^*L423P*^ mutant mice examined in the present study, did also not display significantly elevated blood pressures or serum sodium concentrations, as compared to sex-matched non-mutant control mice. These findings are in line with the results of a previous study [16], where the 24-hrs-urine-volumes, as well as the daily urinary sodium excretion in homozygous *Pou3f3*^*L423P*^ mutant mice were not significantly lower than in non-mutant control mice. The lack of pathogenetically relevant morphological glomerular alterations and the absence of evidence for altered renal sodium homeostasis or volume retention explain why *Pou3f3*^*L423P*^ mutant mice do not display systemic hypertension despite their significantly reduced nephron endowment. The lack of primary pathomorphological glomerular lesions and hypertension in homozygous *Pou3f3*^*L423P*^ mutant mice might actually be advantageous, since it might allow for examination of the effects of reduced nephron numbers independent of concomitant glomerular lesion patterns. This might be particularly important, if *Pou3f3*^*L423P*^ mutant mice are used as a model for a low nephron endowment in experiments analyzing the role of reduced nephron numbers as a predisposing factor for development of aggravated renal/glomerular lesions. Such an experimental approach could comprise the comparison of a group of homozygous *Pou3f3*^*L423P*^ mutant mice and a group of mice with “normal” nephron numbers, both being challenged with an additional renal insult.

In summary, the results of the present study confirm the important function of POU3F3 in nephron patterning, especially in development of the TAL. Additionally, we provide strong evidence that POU3F3 is also involved in nephron induction, the determination of nephron numbers, and in nephron size in the murine kidney. The detailed characterization of the renal morphology and function of *Pou3f3*^*L423P*^ mutants provides the basis for their use as an experimental animal model of low nephron numbers in nephrological research, as well as in further studies examining the molecular and cellular functions of POU3F3 during nephrogenesis.

## Supporting Information

S1 Table Exact p values of statistically analyzed* parameters of homozygous (HOM) and heterozygous (HET) *Pou3f3*^*L423P*^ mutant mice and control (CON) mice at 12 days of age. *Statistically significant differences between genotypes were determined by using a 1-way ANOVA with Gabriel’s post hoc test, differences between male and female mice of the identical genotype by Student’s t-test.
(DOCX)

S2 Table Exact p values of statistically analyzed* parameters of homozygous (HOM) and heterozygous (HET) *Pou3f3*^*L423P*^ mutant mice and control (CON) mice at 60 weeks of age. UACR: Urine albumin-to-creatinine ratio. *Statistically significant differences between genotypes were determined by using a 1-way ANOVA with Gabriel’s post hoc test, differences between male and female mice of the identical genotype by Student’s t-test.
(DOCX)
