# Supplementary material for: Missense Mutation of POU Domain Class 3 Transcription Factor 3 in Pou3f3L423P Mice Causes Reduced Nephron Number and Impaired Development of the Thick Ascending Limb of the Loop of Henle
Source: PLoS One. 2016 Jul 15;11(7):e0158977. doi: 10.1371/journal.pone.0158977 (PMC4946790; doi:10.1371/journal.pone.0158977)
Supplement: S1 Table — *Statistically significant differences between genotypes were determined by using a 1-way ANOVA with Gabriel’s post hoc test, differences between male and female mice of the identical genotype by Student’s t-test. (DOCX) [file pone.0158977.s001.docx]

**S1 Table. Exact p values of statistically analyzed* parameters of homozygous (HOM) and heterozygous (HET) *Pou3f3^L423P^* mutant mice and control (CON) mice at 12 days of age.**

| Parameter | sex | overall | CON vs. HET | CON vs. HOM | HOM vs. HET | *male vs. female* | | |
| --- | --- | --- | --- | --- | --- | --- | --- | --- |
|  |  |  |  |  |  | CON | HET | HOM |
| Body weight | m | 0.025 | 0.999 | 0.074 | 0.028 | 0.5458 | 0.3519 | 0.7445 |
|  | f | 0.086 | 0.998 | 0.104 | 0.097 |  |  |  |
| Kidney weight | m | 0.001 | 0.924 | 0.003 | 0.001 | 0.9625 | 0.7076 | 0.3208 |
|  | f | 0.006 | 0.992 | 0.007 | 0.006 |  |  |  |
| Relative kidney weight | m | 0.003 | 0.623 | 0.040 | 0.019 | 0.6509 | 0.4061 | 0.8708 |
|  | f | 0.110 | 0.999 | 0.014 | 0.010 |  |  |  |
| Urine albumin concentration | m | 0.333 | 0.196 | 0.212 | 0.959 | 0.2085 | 0.4325 | 0.4849 |
|  | f | 0.212 | 0.431 | 0.309 | 0.085 |  |  |  |
| V _(Kidney)_ | m | 0.001 | 0.993 | 0.004 | 0.009 | 0.7104 | 0.6529 | 0.5682 |
|  | f | 0.002 | 0.961 | 0.002 | 0.002 |  |  |  |
| V_V (Cortex/Kid)_ | m | 0.277 | 0.942 | 0.340 | 0.464 | 0.9057 | 0.6368 | 0.5407 |
|  | f | 0.106 | 0.980 | 0.113 | 0.142 |  |  |  |
| V _(Cortex, Kid)_ | m | 0.006 | 0.789 | 0.014 | 0.001 | 0.7458 | 0.9556 | 0.4353 |
|  | f | 0.028 | 0.426 | 0.021 | 0.154 |  |  |  |
| V_V (Med/Kid)_ | m | 0.277 | 0.924 | 0.340 | 0.464 | 0.9057 | 0.9861 | 0.5407 |
|  | f | 0.131 | 0.901 | 0.124 | 0.232 |  |  |  |
| V _(Med, Kid)_ | m | 0.000 | 0.260 | 0.001 | 0.005 | 0.8016 | 0.9527 | 0.8763 |
|  | f | 0.004 | 0.437 | 0.003 | 0.202 |  |  |  |
| V_V (TAL/Kid)_ | m | 0.000 | 0.007 | 0.000004 | 0.000785 | 0.0128 | 0.0551 | 0.9149 |
|  | f | 0.000 | 0.503 | 0.00086 | 0.000023 |  |  |  |
| V _(TAL, Kid)_ | m | 0.000 | 0.085 | 0.000005 | 0.000059 | 0.3168 | 0.1097 | 0.6494 |
|  | f | 0.000 | 0.949 | 0.000299 | 0.000041 |  |  |  |
| V_V (Glom/Kid)_ | m | 0.558 | 0.615 | 0.808 | 0.997 | 0.2478 | 0.9311 | 0.9329 |
|  | f | 0.964 | 1.000 | 0.990 | 0.993 |  |  |  |
| V _(Glom, Kid)_ | m | 0.018 | 1.000 | 0.700 | 0.018 | 0.2938 | 0.8865 | 0.5625 |
|  | f | 0.032 | 0.616 | 0.024 | 0.107 |  |  |  |
| V _(Glom, Kid)_/body weight | m | 0.403 | 1.000 | 0.629 | 0.470 | 0.7057 | 0.0315 | 0.7707 |
|  | f | 0.100 | 0.399 | 0.102 | 0.537 |  |  |  |
| N_V (Glom/Kid)_ | m | 0.187 | 0.533 | 0.190 | 0.650 | 0.8016 | 0.4646 | 0.4318 |
|  | f | 0.036 | 0.464 | 0.027 | 0.174 |  |  |  |
| N _(Glom, Kid)_ | m | 0.000 | 0.031 | 0.000009 | 0.000397 | 0.5073 | 0.2454 | 0.7768 |
|  | f | 0.000 | 0.012 | 0.000006 | 0.000139 |  |  |  |
| N _(Glom, Kid)_/body weight | m | 0.032 | 0.241 | 0.027 | 0.296 | 0.5241 | 0.3856 | 0.8866 |
|  | f | 0.000 | 0.028 | 0.000324 | 0.041 |  |  |  |
| v̅ _(Glom)_ | m | 0.002 | 0.167 | 0.001486 | 0.023 | 0.2478 | 0.6494 | 1.000 |
|  | f | 0.028 | 1.000 | 0.044 | 0.054 |  |  |  |
| v̅ _(Glom)_/body weight | m | 0.565 | 0.632 | 0.786 | 1.000 | 0.2134 | 0.6581 | 0.8185 |
|  | f | 0.811 | 0.996 | 0.949 | 0.874 |  |  |  |

*Statistically significant differences between genotypes were determined by using a 1-way ANOVA with Gabriel’s post hoc test, differences between male and female mice of the identical genotype by Student’s t-test.
