# Supplementary material for: Missense Mutation of POU Domain Class 3 Transcription Factor 3 in Pou3f3L423P Mice Causes Reduced Nephron Number and Impaired Development of the Thick Ascending Limb of the Loop of Henle
Source: PLoS One. 2016 Jul 15;11(7):e0158977. doi: 10.1371/journal.pone.0158977 (PMC4946790; doi:10.1371/journal.pone.0158977)
Supplement: S2 Table — UACR: Urine albumin-to-creatinine ratio. *Statistically significant differences between genotypes were determined by using a 1-way ANOVA with Gabriel’s post hoc test, differences between male and female mice of the identical genotype by Student’s t-test. (DOCX) [file pone.0158977.s002.docx]

**S2 Table. Exact p values of statistically analyzed* parameters of homozygous (HOM) and heterozygous (HET) *Pou3f3^L423P^* mutant mice and control (CON) mice at 60 weeks of age.**

| Parameter | sex | overall | CON *vs.* HET | CON *vs.* HOM | HOM *vs.* HET | male *vs.* female | | |
| --- | --- | --- | --- | --- | --- | --- | --- | --- |
|  |  |  |  |  |  | CON | HET | HOM |
| Body weight | m | 0.001 | 0.989 | 0.0040 | 0.0040 | 0.6200 | 0.1079 | 0.0152 |
|  | f | 0.002 | 0.269 | 0.01511 | 0.065 |  |  |  |
| Kidney weight | m | 0.000 | 0.484 | 0.000065 | 0.000004 | 0.0006 | 0.0014 | 0.0022 |
|  | f | 0.000 | 0.999 | 0.000039 | 0.000048 |  |  |  |
| Relative kidney weight | m | 0.005 | 0.054 | 0.109 | 0.013 | 0.0006 | 0.0200 | 0.0022 |
|  | f | 0.009 | 0.112 | 0.530 | 0.040 |  |  |  |
| Mean blood pressure | m | 0.775 | 0.923 | 0.999 | 0.882 | 0.0369 | 0.7490 | 0.5264 |
|  | f | 0.087 | 0.113 | 0.228 | 0.985 |  |  |  |
| Serum urea | m | 0.002 | 0.997 | 0.0081 | 0.00412 | 0.9487 | 0.4757 | 0.4848 |
|  | f | 0.014 | 0.241 | 0.0415 | 0.0295 |  |  |  |
| Serum cystatin c | m | 0.474 | 0.975 | 0.545 | 0.776 | 0.4020 | 1.000 | 0.6905 |
|  | f | 0.977 | 0.997 | 1.000 | 0.996 |  |  |  |
| Serum sodium | m | 0.822 | 0.998 | 0.905 | 0.948 | 0.1098 | 0.0607 | 0.3358 |
|  | f | 0.454 | 0.994 | 0.536 | 0.668 |  |  |  |
| Urine albumin concentration | m | 0.041 | 0.578 | 0.062 | 0.015 | 0.1320 | 0.1709 | 0.1320 |
|  | f | 0.006 | 0.116 | 0.005 | 0.246 |  |  |  |
| Urine creatinine concentration | m | 0.181 | 0.408 | 0.217 | 0.925 | 0.7009 | 1.000 | 1.000 |
|  | f | 0.012 | 0.676 | 0.013 | 0.132 |  |  |  |
| UACR | m | 0.067 | 0.378 | 0.575 | 0.066 | 0.1905 | 0.3929 | 0.1143 |
|  | f | 0.026 | 0.191 | 0.024 | 0.799 |  |  |  |
| Urine specific gravity | m | 0.002 | 0.999 | 0.00417 | 0.00817 | 0.1373 | 0.5089 | 0.3341 |
|  | f | 0.014 | 0.596 | 0.0141 | 0.0463 |  |  |  |
| V _(Kidney)_ | m | 0.000 | 0.484 | 0.000065 | 0.000004 | 0.0001 | 0.0001 | 0.0001 |
|  | f | 0.000 | 0.999 | 0.000039 | 0.000048 |  |  |  |
| V_V (Cortex/Kid)_ | m | 0.833 | 1.000 | 0.921 | 0.94 | 0.0038 | 0.4484 | 0.3471 |
|  | f | 0.013 | 0.170 | 0.009 | 0.419 |  |  |  |
| V _(Cortex, Kid)_ | m | 0.003 | 0.813 | 0.004 | 0.016 | 0.0006 | 0.0006 | 0.0043 |
|  | f | 0.006 | 0.985 | 0.017 | 0.009 |  |  |  |
| V_V (Med/Kid)_ | m | 0.833 | 1.000 | 0.921 | 0.940 | 0.0279 | 0.5212 | 0.1474 |
|  | f | 0.013 | 0.170 | 0.010 | 0.419 |  |  |  |
| V _(Med, Kid)_ | m | 0.530 | 0.955 | 0.066 | 0.133 | 0.0913 | 0.0040 | 0.0011 |
|  | f | 0.000 | 0.183 | 0.014 | 0.041 |  |  |  |
| V_V (TAL/Kid)_ | m | 0.009 | 0.494 | 0.00798 | 0.07602 | 0.0006 | 0.0003 | 0.0087 |
|  | f | 0.003 | 0.498 | 0.0040 | 0.0376 |  |  |  |
| V _(TAL, Kid)_ | m | 0.006 | 0.981 | 0.0020 | 0.0247 | 0.0379 | 0.0541 | 0.0303 |
|  | f | 0.002 | 0.691 | 0.0030 | 0.0015 |  |  |  |
| V_V (Glom/Kid)_ | m | 0.013 | 0.884 | 0.014 | 0.041 | 0.0001 | 0.0001 | 0.0003 |
|  | f | 0.020 | 0.177 | 0.019 | 0.551 |  |  |  |
| V _(Glom, Kid)_ | m | 0.002 | 0.638 | 0.001576 | 0.009923 | 0.2911 | 0.1993 | 0.9829 |
|  | f | 0.001 | 0.549 | 0.000614 | 0.001013 |  |  |  |
| V _(Glom, Kid)_/body weight | m | 0.029 | 0.302 | 0.025 | 0.351 | 0.7104 | 0.4908 | 0.6623 |
|  | f | 0.343 | 0.677 | 0.387 | 0.934 |  |  |  |
| N_V (Glom/Kid)_ | m | 0.295 | 0.351 | 0.126 | 0.471 | 0.0070 | 0.0022 | 0.0022 |
|  | f | 0.621 | 1.000 | 0.726 | 0.679 |  |  |  |
| N _(Glom, Kid)_ | m | 0.000 | 0.0885 | 0.0001 | 0.0167 | 0.0111 | 0.1893 | 0.1320 |
|  | f | 0.000 | 0.620 | 0.027 | 0.0179 |  |  |  |
| N _(Glom, Kid)_/body weight | m | 0.036 | 0.320 | 0.033 | 0.453 | 0.2086 | 0.4136 | 0.0411 |
|  | f | 0.044 | 0.575 | 0.642 | 0.999 |  |  |  |
| v̅ _(Glom)_ | m | 0.002 | 0.257 | 0.001771 | 0.0150 | 0.0420 | 0.7789 | 0.1775 |
|  | f | 0.002 | 0.215 | 0.000117 | 0.40877 |  |  |  |
| v̅ _(Glom)_/body weight | m | 0.023 | 0.657 | 0.020 | 0.116 | 0.7104 | 0.4908 | 0.6623 |
|  | f | 0.250 | 0.944 | 0.525 | 0.289 |  |  |  |
| V_V (Mes/Glom)_ | m | 0.101 | 0.104 | 0.850 | 0.481 | 0.1231 | 0.4107 | 0.7827 |
|  | f | 0.908 | 0.961 | 0.999 | 0.992 |  |  |  |
| V_(Mes, Glom)_ | m | 0.000 | 0.0033 | 0.000089 | 0.110 | 0.0263 | 0.9426 | 0.2381 |
|  | f | 0.001 | 0.140 | 0.0011 | 0.0478 |  |  |  |
| V_V (Cap/Glom)_ | m | 0.003 | 0.0025 | 0.409 | 0.116 | 0.0054 | 0.5478 | 0.5658 |
|  | f | 0.470 | 0.915 | 0.519 | 0.825 |  |  |  |
| V _(Cap, Glom)_ | m | 0.012 | 0.959 | 0.015 | 0.029 | 0.9015 | 0.3969 | 0.1775 |
|  | f | 0.005 | 0.122 | 0.0039 | 0.177 |  |  |  |
| V_V (Pod/Glom)_ | m | 0.458 | 0.517 | 0.966 | 0.848 | 0.1764 | 0.9069 | 0.5787 |
|  | f | 0.205 | 0.988 | 0.249 | 0.346 |  |  |  |
| v̅ _(Pod, Glom)_ | m | 0.005 | 0.164 | 0.0037 | 0.129 | 0.2136 | 0.4319 | 0.6534 |
|  | f | 0.061 | 0.303 | 0.042 | 0.622 |  |  |  |
| L_V (Cap/Glom)_ | m | 0.458 | 0.517 | 0.966 | 0.848 | 0.1137 | 0.0552 | 0.4422 |
|  | f | 0.205 | 0.988 | 0.249 | 0.346 |  |  |  |
| L _(Cap, Glom)_ | m | 0.000 | 0.939 | 0.00096 | 0.000268 | 0.4098 | 0.2021 | 0.5368 |
|  | f | 0.010 | 0.980 | 0.0136 | 0.0213 |  |  |  |
| L _(Glom-cap, Kid)_ | m | 0.000 | 0.486 | 0.0197 | 0.00121 | 0.7308 | 0.8665 | 0.7922 |
|  | f | 0.019 | 0.896 | 0.0206 | 0.0066 |  |  |  |
| N_V (C/Glom)_ | m | 0.288 | 0.588 | 0.330 | 0.904 | 0.2343 | 0.2180 | 0.0173 |
|  | f | 0.206 | 0.742 | 0.212 | 0.664 |  |  |  |
| N _(C, Glom)_ | m | 0.006 | 0.876 | 0.0082 | 0.0189 | 1.000 | 0.7546 | 0.7922 |
|  | f | 0.01 | 0.132 | 0.0088 | 0.363 |  |  |  |
| N_V (M-E/Glom)_ | m | 0.777 | 0.950 | 0.994 | 0.867 | 0.2824 | 0.1375 | 0.303 |
|  | f | 0.518 | 1.000 | 0.689 | 0.632 |  |  |  |
| N _(M-E, Glom)_ | m | 0.032 | 0.933 | 0.0403 | 0.075 | 1.000 | 0.7546 | 0.7922 |
|  | f | 0.057 | 0.244 | 0.00631 | 0.754 |  |  |  |
| N_V (Pod/Glom)_ | m | 0.071 | 0.721 | 0.069 | 0.259 | 0.5338 | 0.4908 | 0.4286 |
|  | f | 0.249 | 0.428 | 0.349 | 0.991 |  |  |  |
| N _(Pod, Glom)_ | m | 0.000 | 0.543 | 0.000016 | 0.0060 | 0.6116 | 0.8972 | 0.2722 |
|  | f | 0.000 | 0.130 | 0.000165 | 0.0095 |  |  |  |
| v̅ _(Pod)_ | m | 0.091 | 0.376 | 0.092 | 0.647 | 0.1320 | 0.4136 | 0.5368 |
|  | f | 0.869 | 0.963 | 1.000 | 0.950 |  |  |  |
| Th _(GBM)_ | m | 0.022 | 0.821 | 0.109 | 0.024 | 0.4000 | 0.4000 | 0.6286 |
|  | f | 0.007 | 0.079 | 0.129 | 0.030 |  |  |  |
| FSF | m | 0.814 | 0.922 | 0.907 | 1.000 | 1.000 | 1.000 | 0.8571 |
|  | f | 0.648 | 0.734 | 0.571 | 0.376 |  |  |  |
| *RT-qPCR: Pou3f3 vs.Gapdh* relative to CON | m | 0.589 | 0.684 | 0.995 | 0.865 | 0.9930 | 0.3649 | 0.3853 |
|  | f | 0.158 | 0.937 | 0.172 | 0.323 |  |  |  |
| *RT-qPCR: Umod vs. Gapdh* relative to CON | m | 0.009 | 1.000 | 0.018 | 0.014 | 0.9894 | 0.1750 | 0.2204 |
|  | f | 0.027 | 0.066 | 0.046 | 0.925 |  |  |  |
| *RT-qPCR: Pou3f3 vs. Umod* relative to CON | m | 0.000 | 0.673 | 0.00003 | 0.00014 | 1.000 | 0.7244 | 0.0488 |
|  | f | 0.002 | 0.095 | 0.00133 | 0.058 |  |  |  |

UACR: Urine albumin-to-creatinine ratio. *Statistically significant differences between genotypes were determined by using a 1-way ANOVA with Gabriel’s post hoc test, differences between male and female mice of the identical genotype by Student’s t-test.
